# Metatranscriptomic Study of Common and Host-Specific Patterns of Gene Expression between Pines and Their Symbiotic Ectomycorrhizal Fungi in the Genus *Suillus*

**DOI:** 10.1371/journal.pgen.1006348

**Published:** 2016-10-13

**Authors:** Hui-Ling Liao, Yuan Chen, Rytas Vilgalys

**Affiliations:** 1 Department of Biology, Duke University, Durham, North Carolina, United States of America; 2 Division of Infectious Diseases, Department of Medicine, Duke University, Durham, North Carolina, United States of America; INRA Nancy, FRANCE

## Abstract

Ectomycorrhizal fungi (EMF) represent one of the major guilds of symbiotic fungi associated with roots of forest trees, where they function to improve plant nutrition and fitness in exchange for plant carbon. Many groups of EMF exhibit preference or specificity for different plant host genera; a good example is the genus *Suillus*, which grows in association with the conifer family Pinaceae. We investigated genetics of EMF host-specificity by cross-inoculating basidiospores of five species of *Suillus* onto ten species of *Pinus*, and screened them for their ability to form ectomycorrhizae. Several *Suillus* spp. including *S*. *granulatus*, *S*. *spraguei*, and *S*. *americanus* readily formed ectomycorrhizae (compatible reaction) with white pine hosts (subgenus *Strobus*), but were incompatible with other pine hosts (subgenus *Pinus)*. Metatranscriptomic analysis of inoculated roots reveals that plant and fungus each express unique gene sets during incompatible vs. compatible pairings. The *Suillus-Pinus* metatranscriptomes utilize highly conserved gene regulatory pathways, including fungal G-protein signaling, secretory pathways, leucine-rich repeat and pathogen resistance proteins that are similar to those associated with host-pathogen interactions in other plant-fungal systems. Metatranscriptomic study of the combined *Suillus-Pinus* transcriptome has provided new insight into mechanisms of adaptation and coevolution of forest trees with their microbial community, and revealed that genetic regulation of ectomycorrhizal symbiosis utilizes universal gene regulatory pathways used by other types of fungal-plant interactions including pathogenic fungal-host interactions.

## Author Summary

Ectomycorrhizal fungi (EMF) comprise the dominant group of symbiotic fungi associated with plant roots in temperate and boreal forests. We examined host-specificity and gene-expression of five EMF *Suillus* species that exhibited strong patterns of mycorrhizal compatibility/incompatibility with either white pines (*Pinus* subg. *Strobus*) or hard pines (subg. *Pinus*). Using RNA-Seq, we identified conserved transcriptomic responses associated with compatible versus incompatible *Pinus*-*Suillus* species pairings. Comparative metatranscriptomic analysis of compatible vs. incompatible pairings allowed us to identify unique sets of fungal and plant genes associated with symbiont recognition and specificity. Comparativ transcriptomic study of the *Suillus*-*Pinus* system provides insight into the core functions involved in ectomycorrhizal symbiosis, and the mechanisms by which host-symbiont pairs recognize one another.

## Introduction

Growing evidence has shown that many symbiotic plant-microbial associations including pathogenic as well as mutualistic symbioses are governed by similar genetic interaction mechanisms [1,2]. For example, in many groups of pathogenic fungi and oomycetes, coevolution with their plant hosts has resulted in typical 'arms-race' patterns of interactions, in which pathogens evolve batteries of effectors that suppress plant defense responses, while plants evolve modified receptors that sense microbial molecules and reactivate plant defense responses [3]. The molecular functions of several fungal and oomycete effectors involved in host-pathogen recognition have recently been elucidated. For instance, cysteine-rich avirulence genes (*Avr*) have been identified in several fungi including *Cladosporium fulvum* and *Melampsora lini* [4, 5], while *Avr1b* was isolated from the oomycete *Phytophthora sojae* [6]. Studying the functions of these effectors is a challenging task, because of the highly divergent nature of effectors in diverse taxa of pathogenic microbes and the lack of similarity of the sequences of these effectors to other proteins in public databases. Plant defense proteins that perceive microbial effectors include nucleotide-binding leucine-rich repeat (NB-LRR) proteins [1, 7, 8] and cell membrane receptors (e.g. phosphatidylinositol 3-P) [9]. These receptors can be activated by direct binding of effectors or modified by effector-associated proteins, leading to a plant-defense response.

Mutualistic plant-fungal interactions, including arbuscular mycorrhizae and ectomycorrhizae, also share similar conserved genetic interaction mechanisms with other symbiotic plant-fungal systems [10–12]. Over 30 plant families are known to form ectomycorrhizal associations with over 80 lineages (250 genera) of fungi [13]. A highly diverse community of EMF form the dominant guild of soil microbes in most of the world's forests [14,15], where they provide their plant hosts with essential resources (N, P, H_2_O) as well as protection from pathogens, in exchange for photosynthetically fixed carbon [16].

Details about molecular interactions between EMF and their plant hosts are emerging. Recent studies have identified differentially expressed genes associated with EMF symbiosis for several EMF-plant interactions including *Pisolithus microcarpus* with *Eucalyptus* [17], *Paxillus involutus* with *Betula* [18], and *Laccaria bicolor* with different *Populus* spp. [2]. One of these genes, a small secreted protein (MiSSP7) produced by the ectomycorrhizal basidiomycete *Laccaria bicolor*, functions as a critical effector for compatible mycorrhizal interaction with *Populus*. MiSSP7 was shown to be imported into plant nuclei where it suppresses plant host defenses, enabling mycorrhiza formation. Other recent studies also demonstrated that jasmonic acid (JA) and related plant defense-activated compounds are produced by *Populus* in response to signals from their symbiont [19,20]. These results suggest a general involvement of JA-mediated and other conserved plant signaling pathways for plant-fungal communication during EMF symbiosis. Similar to the mechanisms of EMF interaction in *Laccaria* [2], plant pathogenic fungi (e.g. *M*. *larici*) can also deliver SSPs to multiple cellular compartments in *Populus* [21]. These studies demonstrate that EMF are able to modulate plant defense system during symbiosis [2,10,21], and suggest that that most plant-microbial associations (including pathogenic and mutualistic interactions) may be governed by similar mechanisms. Unlike biotrophic/necrotrophic parasitisms, mutualistic fungal-plant interactions such as EMF must also establish stable long-term relationships with their living host cells, with benefits to both the fungus and its host. Thus, there is considerable potential for an array of distinct elements to regulate the host-specific communications of symbiosis compared to plant-pathogen interactions.

Many groups of EMF are known to exhibit preference or specificity for different plant host genera [22,23]. A good example of strong host-specificity is the bolete genus *Suillus*, which grows in association with the conifer family Pinaceae [24,25]. Most species of *Suillus* form ectomycorrhizae with specific Pinaceae host species (e.g., white pine, douglas fir, larch), suggesting a long history of plant-fungal coevolution in this genus [26–28]. Other examples of EMF with host-specific interactions include *Laccaria bicolor*, which shows differential host-compatibility with different species of *Populus* [29], and *Paxillus involutus*, which favors *Betula* as a host over *Populus* [30]. In order to study the molecular basis for host-specificity between different *Pinus* and *Suillus* species, we used pairwise plant-fungal bioassays to identify patterns of compatible and incompatible EMF interactions. Compatible EMF interactions are characterized by morphogenesis of plant and fungal tissues leading to development of modified plant short roots with bifurcated root tips that are sheathed by a hyphal mantle over the root epidermal surface, with hyphal ingrowth into the root cortex to form the Hartig-net [31]. In contrast, incompatible EMF interactions fail to induce root morphogenesis, resulting in little or no mycelial growth, and are morphologically indistinguishable from uninoculated (non-symbiotic) roots.

The pace of genetic studies of EMF-plant symbiosis has greatly accelerated by expanding numbers of genome sequencing for many EMF [10]. Though study of most EMF is still hindered by a lack of ‘finished’ genomes, we recently developed a procedure that employs RNA-Seq and de-novo assembly and annotation to characterize the metatranscriptome of EMF associated with *Pinus taeda* from field-collected mycorrhizal root clusters [32]. Here we apply metatranscriptomic profiling to study compatible versus incompatible mycorrhizal interactions from both plant and fungal perspectives. Our studies demonstrate that *Suillus* and *Pinus* each exhibit well-differentiated transcriptomic profiles during compatible and incompatible interactions. Comparison of expression patterns in compatible and incompatible pairings helped us to identify gene sets associated with plant-fungal recognition and establishment of EMF symbiosis.

## Results

### Host-specific relationships between *Suillus* and *Pinus* species

To investigate occurrence of *Suillus* in natural Pinaceae forests, we first examined patterns of host specificity for *Suillus* operational taxonomic units (OTUs) detected by a recent survey of North American pine forest soils using next generation amplicon sequence analysis of the ribosomal RNA internal transcribed spacer (ITS) region [14]. Eleven *Suillus* OTUs detected by that survey (out of a total of >10,000 fungal OTUs detected across North America) exhibit distinct host range patterns corresponding with different Pinaceae hosts (S1 Fig): *S*. *glandulosa with Picea glauca; S*.*hirtellus* and *S*. *cothurnatus* with *Pinus taeda; S*. *granulatus*, *S*. *spraguei (*= *S*. *pictus)* and *S*. *americanus* with *Pinus strobus;* and an unidentified *Suillus* sp. with *Pinus monticola*. Several *Suillus* species were observed to be broadly associated with multiple *Pinus* species, including *Suillus brevipes*, which is associated with several *Pinus* spp. across North America (S1 Fig) but was restricted to hosts in the subgenus *Pinus* (*P*. *ponderosa*, *P*. *contorta*, *P*. *banksiana*, and *P*. *taeda)*.

**Fig 1:**
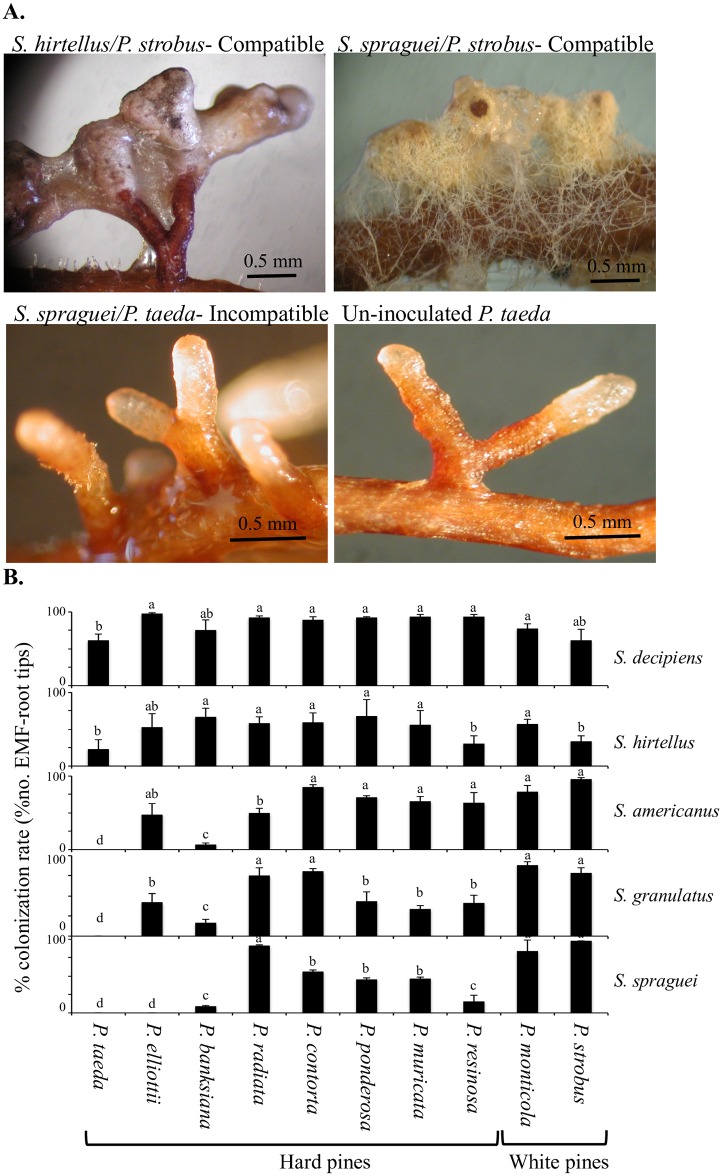
Ectomycorrhizal compatibility and incompatibility between *Suillus—Pinus* species pairings. **(A)** During a compatible EMF interaction, *Suillus*-inoculated roots develop characteristic ectomycorrhizas with short swollen (bifurcated) root tips with well-developed hyphal sheath and Hartig-net (observed in cross-sections of root tips, S1 Fig). Incompatible EMF interactions fail to establish mycorrhizae, with little or no fungal colonization, and are morphologically indistinguishable from un-inoculated (non-mycorrhizal) control roots (also shown). **(B)** Ectomycorrhizal compatibility between different species of *Suillus* and *Pinus* measured as a proportion of EMF root tips versus total bare root tips (n≥3). Tukey test was used to test significance across *Pinus* species within a *Suillus* species (P<0.05). Means marked by the same letters were not significantly different. The complete list of fungal cultures and spore prints used is listed in S3 Table.

To study host specificity, a plant bioassay was developed using axenically grown pine seedlings inoculated with *Suillus* basidiospores to establish *Suillus-Pinus* mycorrhizae *in vitro* [31]. Seedlings of ten *Pinus* species were inoculated in all pairwise combinations with basidiospores of five *Suillus* species and scored for ectomycorrhiza formation after 8 weeks growth. In *Pinus*, successful formation of ectomycorrhizae (compatible interaction) results in a series of characteristic morphogenetic changes to young root tips that become swollen and bifurcated, and ensheathed by a mycelial mantle which penetrates into the root cortex to form a Hartig net [33] (Fig 1A and S2 Fig). In contrast, incompatible pairings are characterized by little or no colonization of roots by fungal mycelium (both fungal mantle and Hartig-net absent).

Basidiospore inoculations of two generalist species, *S*. *hirtellus* and *S*. *decipiens*, resulted in well-developed (compatible) ectomycorrhizae with most *Pinus* species (Fig 1B), when *S*. *hirtellus* had relatively lower rates of colonization on all hosts. Three white pine specialists (*S*. *granulatus*, *S*. *americanus* and *S*. *spraguei*) readily formed ectomycorrhizae with white pines (*P*. *strobus* and *P*. *monticola)*, but had lower colonization rates on hard pines (e.g. *P*. *banksiana*), and failed to form visible ectomycorrhizae on *P*. *taeda* (incompatible pairing) (Fig 1B).

### Transcriptomic activity of *Suillus* and *Pinus* mycorrhizal roots

Variation in mycorrhizal compatibility between different *Suillus* and *Pinus* species suggests that genetic differences underlie host recognition and specificity during ectomycorrhizal symbiosis. To test this hypothesis, we compared transcriptomic activities across a panel of compatible and incompatible root tip samples formed by inoculation of three *Pinus* species (*P*. *monticola*, *P*. *strobus*, *P*. *taeda*) with four species of *Suillus* (*S*. *americanus*, *S*. *granulatus*, *S*. *spraguei*, and *S*. *decipiens*). Detailed descriptions of the individual *Suillus-Pinus* sample pairs, including strains used are listed in S1 Dataset. Transcriptomes from uninoculated pine roots were included as controls (to confirm that *Suillus* genes were not expressed by uninoculated roots) along with pure cultures of each fungal species (as references for transcriptome assembly). Comparative transcriptome profiling was used to identify candidate genes involved in *Pinus*-*Suillus* recognition (Table 1). The computational strategies included a) *de novo* transcriptome assembly to identify reads representing genes for different rRNA, *Suillus*, *Pinus*, and b) comparative transcriptomic analysis to identify common (core) and unique (host-specific) genes involved in symbiosis (see Materials and Methods, and SI text A1-A4; S3–S5 Figs). Unique genes were defined as upregulated genes detected in the RNA contig assembly of one *Suillus* species, but absent in other species examined. However, whether these genes are truly unique to different *Suillus* species still need to be determined through whole genome sequencing.

**Table 1:** Compatible and incompatible Suillus-Pinus species pairings used for comparative transcriptomic analysis. For each *Suillus-Pinus* species pair, spore prints from two to three different fruit bodies (biological replicates) were used to inoculate *Pinus* seedlings. Collection data with source information for *Suillus* cutures, spore prints (and voucher specimens) are listed in S1 Dataset & S3 Table.

	*S*. *americanus*	*S*. *granulatus*	*S*. *spraguei*	*S*. *decipiens*
*P*. *monticola*	Compatible interaction. ID of spore prints used: SA0005; SA0010; SA0011	Compatible interaction. ID of spore prints used: SG0004; SG0009; SG0014	Compatible interaction. ID of spore prints used: SS0006; SS0012; SS0013	Compatible interaction. ID of spore prints used: SD0002; SD0003; SD0008
*P*. *strobus*	Compatible interaction. (Sa/Ps rep1 to rep3) ID of spore prints used: SA0005; SA0010; SA0011	Compatible interaction. ID of spore prints used: SG0004; SG0009	Compatible interaction. ID of spore prints used: SS0006; SS0012; SS0013	Compatible interaction. ID of spore prints used: SD0002; SD0003; SD0008
*P*. *taeda*	Incompatible interaction. ID of spore prints used: SA0005; SA0010	Incompatible interaction. ID of spore prints used: SG0004; SG0009	Incompatible interaction. ID of spore prints used: SS0006; SS0012	Compatible interaction. ID of spore prints used: SD0002; SD0003; SD0008

**Fig 2:**
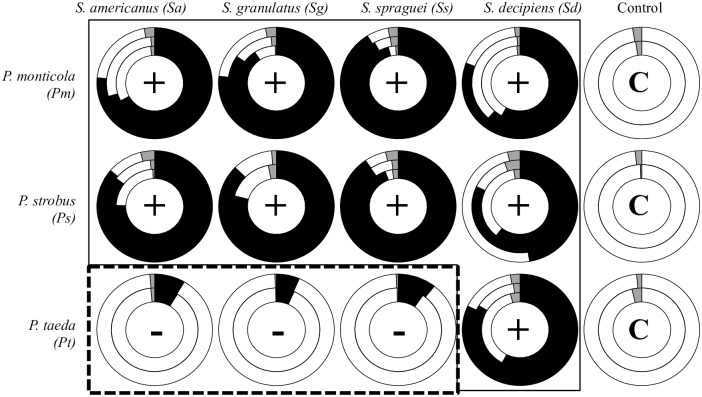
Proportion of metatranscriptomic RNASeq reads assigned to *Suillus* (black), *Pinus* (white), or other fungi (grey) during compatible (+) vs. incompatible (-) EMF interactions. Nested circles within graph represent individual pine seedlings (biological replicates) inoculated with basidiospores of different *Suillus* species. Controls (C) are uninoculated *Pinus* roots. Details with read numbers and gene annotations are shown in S1 Dataset.

Up to 28 million (M) high quality reads were recovered from inoculated root tips using RNA-Seq (approx. 1 mg root tissue per sample, equal to about ten root tips) (S1 Dataset). Compatible *Pinus-Suillus* pairs resulted in roughly equal numbers of plant and fungal reads, while incompatible pairs resulted in much lower number of fungal reads compared to the corresponding plant reads (Fig 2). These differences of *Suillus/Pinus* reads recovered from compatible and incompatible interactions are also consistent with to the higher proportion of fungal biomass present in compatible versus incompatible mycorrhizal pairings. The *Suillus* transcriptome generated from *de novo* assembly of pooled data was used to identify 15M (51% of total reads) and 2M (6.1% of total reads) reads from compatible and incompatible reactions, respectively (Fig 2 and S1 Dataset). Approximately 3M (11% of total reads) and 21M (66% of total reads) *Pinus* transcriptome reads were also recovered from compatible and incompatible pairings, which could be matched to 44% and 69% of publicly available *Pinus* EST databases (~0.3M ESTs), respectively. In total, 11,029 and 5,947 *Suillus* contigs were obtained through de novo assembly from compatible and incompatible root samples respectively (S1 Dataset).

### Expression of *Suillus* genes specific for different species of *Pinus*

**Fig 3:**
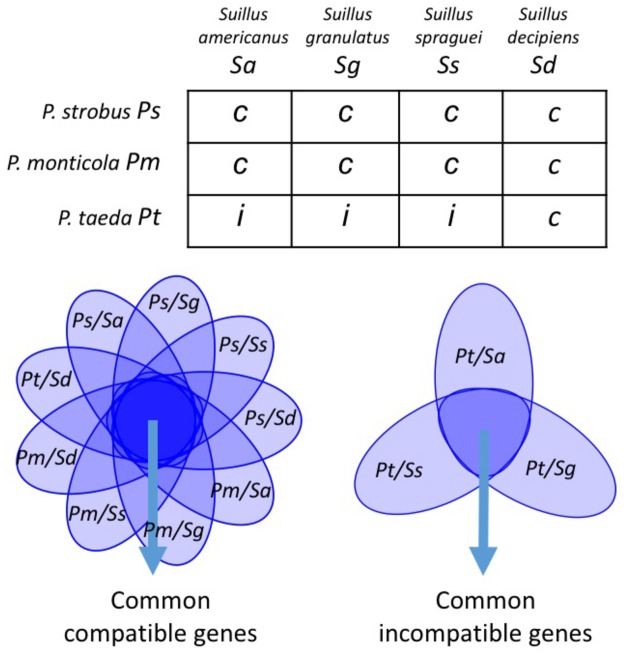
Experimental design showing compatible (c) and incompatible (i) mycorrhizal pairings used for RNASeq analysis to identify common compatible and common incompatible gene sets. After de novo assembly and annotation, common and unique compatible/incompatible gene sets are identified for each species pair of mycobiont (*Suillus*) and phycobiont (*Pinus*).

**Fig 4:**
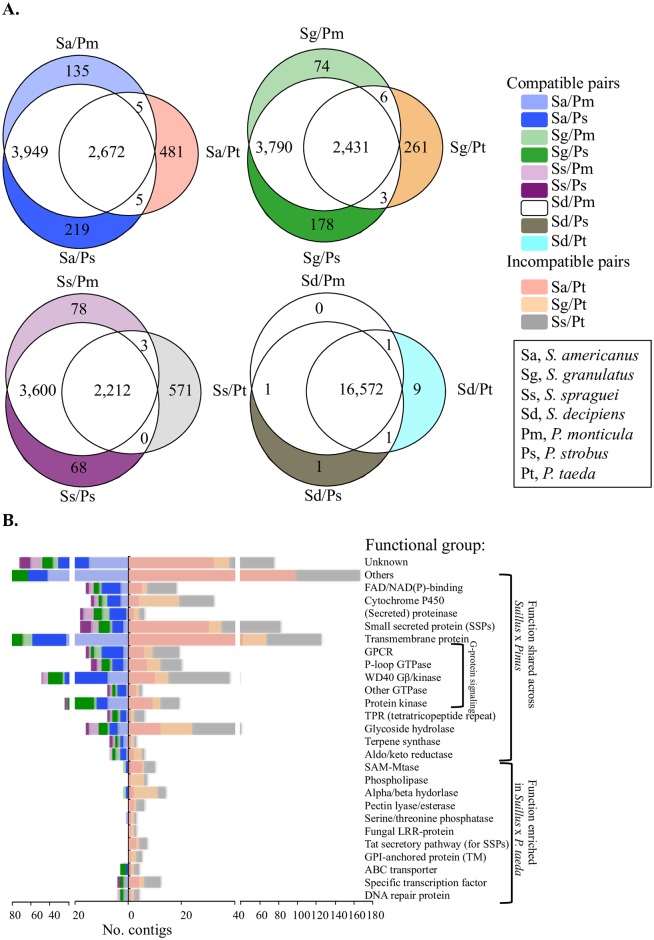
Unique and common *Suillus* genes that are upregulated in response to different pine hosts. (A) Venn diagrams illustrating number of shared and unique genes for 4 *Suillus* species interacting with 3 *Pinus* hosts. Color backgrounds indicate normalized counts of upregulated host-specific transcripts (“unique genes”) identified from the individual pair combinations. Color coding is same for panels A and B. False discovery rate of 5% was used to identify unique genes with at least twofold change in expression (n = 3 for the compatible pairs and n = 2 for incompatible pairs). (B) Color-bar graph showing normalized functional categories of upregulated genes expressed during compatible and incompatible interactions (from Fig 5A). Abbreviations used for *Suillus* and *Pinus*: *S*. *americanus* (Sa), *S*. *granulatus* (Sg), and *S*. *spraguei* (Ss).*P*. *monticola (Pm)*; *P*. *strobus (Ps)*; *P*. *taeda (Pt)*.

We hypothesized that pairings between different *Suillus* (and *Pinus*) species would share common gene expression patterns during compatible vs. incompatible pairings. Similarly, unique gene sets expressed by individual *Suillus/Pinus* pairings could also be identified (Fig 2). Here we defined “common genes” as the core sets of genes that were upregulated (> 2-fold) in response to compatible hosts; in contrast, “unique genes” were identified as those were only expressed in individual *Suillus* spp. in response to specific *Pinus* host species. To test these hypotheses, we used comparative transcriptomic analysis to identify *Suillus* and *Pinus* expressed genes during compatible and incompatible ECM interactions of four *Suillus* species grown with three different hosts, *P*. *monticola*, *P*. *strobus*, and *P*. *taeda*, (Fig 3). To compare gene expression patterns between interacting fungal and host genomes, sequencing reads aligned to either *Suillus* or *Pinus* contigs were normalized using DESeq package (ver. 1.14.0) [34]. (Details were provided in Support Information SI A2, S5 Fig). Gene expression biplots revealed strong differences between compatible and incompatible EMF pairings (S6A and S6B Fig). All of the compatible EMF pairings showed similar expression patterns of *Suillus* genes, even on different hosts (e.g. *P*. *strobus* and *P*. *monticola*) (S7 and S8 Figs), which suggests that different *Suillus* species all employ common regulatory pathways across different compatible host species. Significant differences were observed in gene expression between compatible and incompatible reactions (t-test, p-value < 0.01) (Fig 4). On average, 8,765 *Suillus* contigs were upregulated when they grew with compatible hosts, whereas fewer contigs (1,918 contigs in average) were upregulated from incompatible pairings (S1 Dataset).

Gene expression patterns were analyzed among all individual *Suillus*-*Pinus* species pairs to identify common genes involved in both compatible and incompatible interactions (SI text A2; S5 Fig). A majority of *Suillus* transcripts (~3,800 contigs) were similarly regulated in response to different compatible *Pinus* species. We compared the sequence identities of these genes across all four *Suillus* species and identified 231 “common genes” that were upregulated during the compatible mycorrhizal interactions (Fig 3; SI text A3; S1 Dataset). In contrast to common genes expressed during compatible interaction, a smaller number of genes (261–571 genes) were found to be upregulated during incompatible interactions in different *Suillus* species (Fig 4A). BLASTX search against all four *Suillus* species only identified seven common genes expressed during incompatible mycorrhizal interactions in all species (S1 Dataset). Functional annotations of these seven common genes identified two GHs (glucoside hydrolase), one F-box, one fatty acid desaturase, one signal transduction receptor, and two genes with unknown functions.

### Core functions of *Suillus* genes involved in host recognition

**Fig 5:**
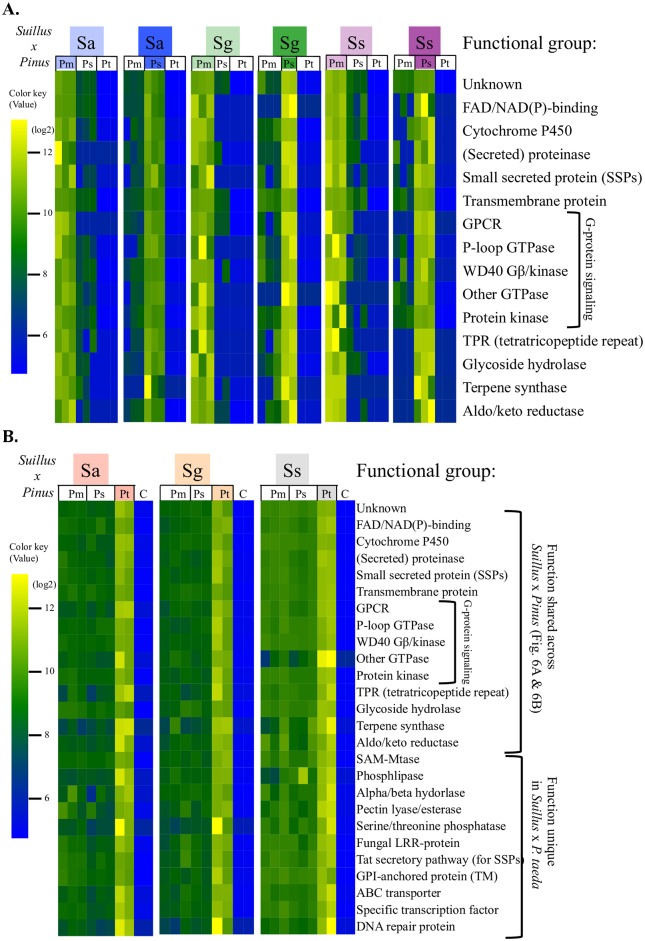
Comparative gene expression of *Suillus* genes expressed with different *Pinus* hosts during compatible (Fig 6A) and incompatible (Fig 6B) mycorrhizal interactions. *Suillus* genes identified from Fig 4 were grouped according to function and relative expression rate (SI text A4), and plotted as a heatmap (color coding same as in Fig 4). *Suillus/Pinus* species pairs with over 500 unique genes are shown in Fig 4A. Only 10 unique genes were identified from *S*. *decipiens/Pinus* pairs. These include 2 SSPs, 1 P-loop, 1 WD40 and 6 unknown; their annotations are provided (with other 3 *Suillus* spp. in S1 Dataset. Significance was determined by normalization of reads across pairings (using DESeq) with false discovery rate (FDR) of 5% using Benjamini-Hochberg test to identify highly expressed transcripts with at least 2-fold change. The color key shows log2 fold changes of the normalized read number. Gene expression in uninoculated *P*. *taeda* roots (“C”) is also shown. Complete read count data for all genes and treatments are shown in S1 Dataset.

In contrast to sharing of 231 expressed genes in compatible mycorrhizal interactions, most genes associated with incompatibility were unique to individual *Suillus-Pinus* species pairs. These included a large number of SSPs, G-proteins, and other genes with little similarity/homology to each other or with other known genes, suggesting that these unique genes for host specificity are highly diverse at the genomic level (Fig 4A, for detailed analysis strategies see SI text A4 and S5 Fig). Unique genes varied among different plant-fungus combinations (from 68 to 571 genes for an individual pair Fig 4B), and were found to represent 14 functional groups (Fig 4B) with similar functions but very low sequence similarity to one another (S1 Dataset). Over two thirds of unique genes expressed by *Suillus* spp. were related to G-protein signaling, such as G-protein coupled receptor (GPCR), GTPase P-loop, Gβ WD40, and G-protein regulated kinases (Figs 4B and 5), which suggests a strong involvement of G-protein pathways in host-specific recognition. Other differentially expressed *Suillus* genes were those related to FAD/AND(P) binding, cytochrome P450-related, secretory, catalysis (proteinase/hydrolysis/reductase/terpene synthesis) and nucleus-associated genes.

Of the 261–571 contigs that were strongly upregulated in response to *Suillus-Pinus* incompatibility (Fig 5A), functional profiling revealed 22 to 28 contigs for shared functions related to *tat* signaling pathway for exporting small secreted proteins, GPI anchored proteins, fungal LRR-domain proteins, phosphatase, and pectin lyase (Figs 4B and 5B). Expression of these genes was not detected in most compatible pairings.

### Putative *Suillus* effectors for host recognition

**Fig 6:**
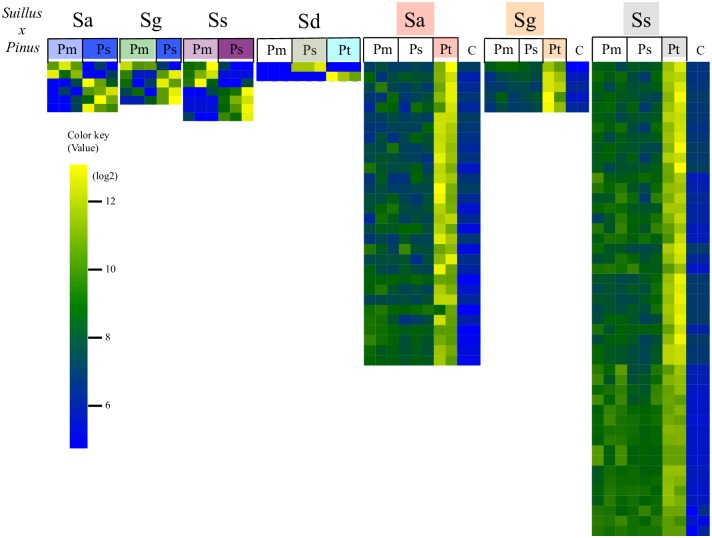
Expression of unique *Suillus* small-secreted proteins (SSPs) during compatible EMF interactions with *Pinus*. Heatmap shows normalized gene expression of SSPs for individual *Suillus* spp. (Sa, Sg, Ss, or Sd) paired with different *Pinus* spp. Each gene was significantly overexpressed in one of the pair combinations as determined by comparisons with FDR<0.05 using Benjamini-Hochberg test. Gene expression in uninoculated *P*. *taeda* roots (“C”) is also shown. See S1 Dataset for complete gene annotations and read counts.

Fungal small-secreted proteins (SSPs) are predicted to be key mycorrhizal effectors for the recognition of EMF by their plant host system. Using domain analysis (SI text A4), SSPs were defined by several criteria including (a) size smaller than 300 amino acid, (b) signal peptide predicted at the N-terminal and extracellular localization activity; (c) absence of transmembrane domains; (d) absence of endoplasmic reticulum retention motifs [12]. 47 *Suillus* SSP's matching these criteria were upregulated in response to different *Pinus* hosts (Fig 6). More SSPs were upregulated during incompatible than compatible interactions. At the sequence level, most SSPs are highly diverse and do not share sequence similarity with other SSPs from currently available databases. Most *Suillus* SSPs were also observed to be highly diverse in their tertiary structure (S8A Fig).

### Unique genes of *Pinus* associated with *Suillus* recognition

Comparative transcriptional profiling of *Pinus* genes across the *Suillus*-root pairs also identified a large number of pine transcripts with similar expression in response to compatible vs. incompatible EMF pairings (~18,000 contigs; S10 Fig). Overall, a smaller number of *Pinus* genes (from 253–5452 contigs) were differentially expressed in response to pairings with different species of *Suillus*. The largest number of upregulated genes was observed for *Pinus*-*S*. *spraguei* interactions compared to other compatible pairs, suggesting the possibility of a greater *Pinus* response to *S*. *spraguei*.

**Fig 7:**
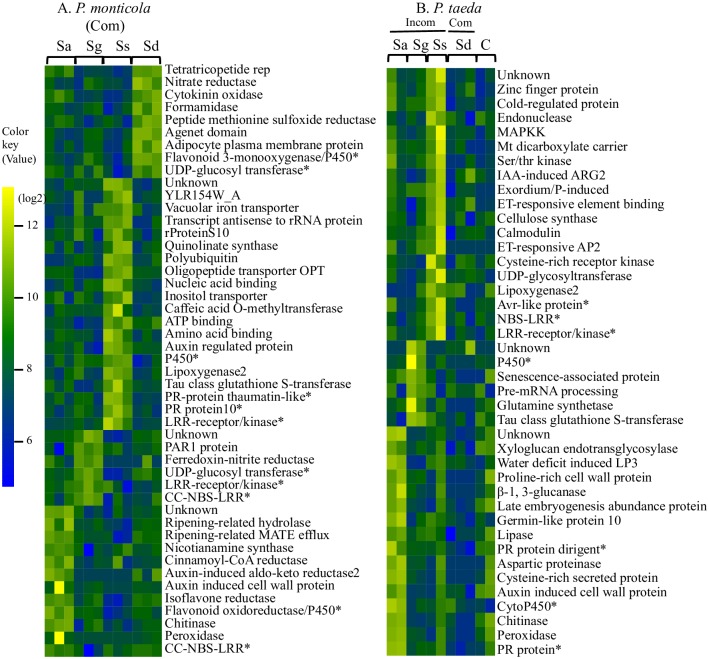
Relative expression of *Pinus monticola* (A) and *Pinus taeda* (B) functional gene groups in response to individual *Suillus* species (Sa, Sg, Ss and Sd). Gene expression in uninoculated *P*. *taeda* roots (“C” in Fig 7B) is also shown. Significance was determined using normalized read counts with FDR<0.05 using Benjamini-Hochberg test. Unique functional genes shared across all pairings are marked by an asterisk. Unique *Pinus* genes were further characterized by functional annotation (SI text A4).

Highly expressed *Pinus* genes with at least two-fold change (FDR<0.05) were further characterized as “pine unique genes” involved in fungal recognition (expressed by individual *Pinus* spp. in response to specific species of *Suillus*) (Fig 7). On average, 20 *Pinus* contigs were identified as unique genes for every *Suillus*-pair sample. BLASTX annotation identified sets of unique *Pinus* genes with common function involved in *Suillus* recognition, including genes for leucine rich (LRR)- proteins, UDP-glucosyl transferase, and cytochrome P450. Inoculation with *S*. *spraguei* also upregulated distinct *Pinus* genes encoding lipoxygenase 2, suggesting a potential effect on JA pathways for the *Pinus*-*S*. *spraguei* interaction.

Two different sets of *P*. *taeda* genes were found to be expressed during incompatible response including fungal species-specific (Fig 7) and species-nonspecific genes (S11 Fig). Comparative transcriptomic analysis also captured changes in expression patterns of 460 *P*. *taeda* genes associated with incompatibility, but these do not appear to be *Suillus* species-specific (S11A Fig). A number of *Pinus* genes known to be associated with defense responses were only weakly or not expressed in compatible pairings and uninoculated roots, including genes involved in plant resistance and water stress response including genes for salicylic acid acquired resistance (NDR1), ethylene-responsive transcription factor and RNA helicase, leucine rich proteins (e.g. Cf2.1, receptor kinase), thaumatin-like proteins, dehydrin and water deficit induced-LP3 (S11B Fig).

## Discussion

Comparative metatranscriptomic profiling of compatible vs. incompatible *Pinus-Suillus* interactions reveals several novel aspects of ectomycorrhizal symbiosis: (a) *Suillus* are transcriptionally active under both compatible and incompatible reactions; (b) *Suillus* spp. vary in their host specificity with different species of *Pinus*; (c) Suillus spp. share common sets of genes expressed during compatible and incompatible responses with different Pinus spp.; (d) Individual *Pinus-Suillus* species pairings induce expression of unique gene sets including genes for small secreted proteins (SSPs)/G-protein signaling pathway (*Suillus* genes) and LRR/PR proteins (*Pinus* genes).

We hypothesize that the shared functions among “common genes” contribute to a common role in core mechanisms of host-recognition. In contrast, “unique genes” may be involved in recognition between individual *Suillus-Pinus* species pairs. During incompatible interactions, these unique genes are largely associated with host recognition, specificity, and incompatibility.

### Common genes of *Suillus*

We identified 231 common genes expressed during compatible mycorrhizal interactions (9 out of 12 *Suillus-Pinus* species pairings, Fig 3). In contrast, comparative analysis revealed only 7 common genes expressed during incompatible interactions between all three white-pine specialists when paired with loblolly pine (*P*. *taeda*). These findings suggest that different *Suillus* spp. share a common set of genes involved in compatible but not in incompatible responses. These estimates are likely to be higher, however, since our strategy employing de novo assembly and annotation could not detect less abundantly expressed genes without much deeper sequencing or access to a high quality reference genome. Further mapping of compatible/incompatible gene sets to fully-sequenced reference genomes of *Suillus* and *Pinus* is likely to reveal additional shared common genes involved in compatible/incompatible interactions.

#### Host-specific EMF interactions between Pinus and Suillus utilize conserved gene regulatory pathways

We were able to identify several sets of “unique genes” expressed by individual *Suillus-Pinus* species pairs. Most of these belong to gene regulatory networks associated with host-recognition, and include SSPs and G-proteins of *Suillus*, and LRR proteins of *Pinus*. During pathogenic fungal-plant interactions, fungal SSPs are recognized with high specificity by plant LRR-protein receptors [35]. Most *Suillus* SSPs that we detected are species-specific and lack sequence similarity to other known proteins. In plants, LRR-containing R proteins are able to recognize unique SSPs produced by their fungal pathogens [36,37]. Our study provides evidence for the involvement of SSP-LRR interactions during ectomycorrhizal symbiosis between *Suillus* and *Pinus*. Up-regulation of unique fungal SSP and plant LRR genes suggests that EMF and their plant hosts utilize a similar recognition system (SSP-LRR recognition) for species-specific interaction as other pathogenic fungal-plant interaction [6,7,38]. In addition to expression of SSP-LRR genes, upregulation of *Pinus* genes for jasmonic acid/ethylene (JA/ET) pathways during incompatible interactions (Fig 7 and S10B Fig) also suggests the involvement of these pathways in EMF symbiosis. Interestingly, expression of genes for salicylic acid (SA) mediated pathway that is associated with plant-defense in other hosts such as *Populus* [39] were not observed during EMF compatibility between *Suillus* and *Pinus*.

Fungal G-protein pathways are predicted to have a number of important roles including mating compatibility and pathogenicity [40,41]. Recent evidence for expansion of gene families for WD40-domain proteins and GTPase α in *L*. *bicolor* [42,43] also suggests the involvement of G-protein pathway in EMF symbiosis. Detailed characterization of several representative unique genes of *Suillus* and *Pinus* are illustrated below.

#### Unique genes of *Suillus*

Comparative transcriptomics allowed us to identify several novel *Suillus* genes involved in EMF symbiosis, including both common as well as unique genes which may share similar functions (e.g. small secreted proteins, G-protein signalling). Coexpression of multiple common and unique genes during EMF-plant symbiosis suggests that many of the unique genes collectively contribute to host specificity. Involvement of multiple unique genes from an individual functional group might explain how species of *Suillus* exhibit different degrees of host specificity. Gain or loss of function of individual unique genes for *Suillus* might also result in host-range expansion or restriction. Further studies are necessary to confirm the function of these genes during EMF symbiosis.

In addition to their role in host-specificity, structural analyses suggest a diversity of functional roles for SSPs in *Suillus*. None of the SSPs we detected had importin α-dependent nuclear localization signals which are necessary for import into the plant nucleus. Many *Suillus* SSPs do contain a N-glyco motif indicating their role in extracellular activity and cell-cell interactions (examples shown in S9A Fig). Several SSPs identified from *S*. *granulatus* were found to contain a short N-terminal motif, RXLR, which has been reported involved in host-cell translocation and phosphatidylinositol 3-phosphate binding [44]. Based upon tertiary structure prediction, most of the *Suillus* SSPs show no significant sequence similarity to proteins of known structure using protein prediction tools (Phyre2 and I-TASSER) (examples shown in S9A Fig). Many *Suillus* SSPs do contain one or two L-shaped alpha-helix folds (examples shown in S9A Fig) similar to the structure observed in some avirulence (Avr) genes [45]. L-shaped alpha-helical structures are asymmetric, and are found in diverse proteins that are involved in protein-protein interaction [46]. These characteristics suggest internalization of SSPs into plant cells. Overall, most SSPs identified from *Suillus* appear species-specific, diverse in their sequence structure, and lacking sequence similarity to known proteins, making it difficult to characterize their function. Further study is needed to understand the molecular basis of SSP function in these mycorrhizal plant systems.

There is a clear requirement for G-protein signaling in host-recognition by *Suillus*. Over 100 contigs, more than 40% of *Suillus* “unique genes” in each pair, encode proteins associated with G-protein transduction, including G-protein coupled receptor (GPCR)-like proteins, heterotrimeric GTPases, and kinases. GPCRs, the cell surface proteins, enable *Suillus* to respond to a variety of extracellular cues and transmit the signals through Gα/Gβγ, and activate downstream effectors. Besides GPCR, a large proportion of gene counts (over 15% of “unique genes”, representing approximately 30 contigs for each pair) were detected with alpha helix transmembrane domains that are likely act as host-specific receptors (Fig 4B and S9B Fig). Although we could not predict which kind of signals these membrane proteins detect, the redundancy of their protein structures in different *Suillus* spp. (SI text A5, S9B Fig) suggests their potential to perceive a structurally diverse set of compounds released from specific hosts at the symbiotic interface.

Possible downstream responses triggered by G-protein pathways are still unclear. Several “unique genes” for nucleus activities (e.g. DNA helicase and specific transcriptional factors) were identified across different pair samples, hinting at the possibility that G-protein pathways are involved in transcriptional control of EMF symbiosis. In addition to genes for signal transduction, we also identified “unique genes” that regulate enzyme activities of FAD/NAD(P) binding, cytochrome P450, proteinase, glycoside hydrolase and terpene synthase, indicating involvement of certain metabolic pathways in host adaptation. It is unclear whether those enzymes are also regulated by G-protein mediated signaling.

#### Unique genes of *Pinus*

At least 20 fungal-specific gene groups were identified from different *Pinus* spp. (Fig 7). Most of these genes encode proteins known to be directly or indirectly involved in plant defense responses (S1 Dataset). Based on nucleotide sequences, distinct genes for plant LRR-proteins were strongly upregulated in response to inoculation with different species of *Suillus*, suggesting their function in mediating EMF recognition. These LRR-containing proteins include receptor-like kinase or CC-NBS type, often referred to as “R” (resistance) proteins that mediate recognition of fungal effectors. Our study identified several species-specific LRR-proteins in *Pinus* that are involved in compatible and incompatible reactions. In addition, a number of enzymes for metabolites (e.g. flavonoids) were uniquely identified in *Suillus-Pinus* specific pairs. It is unclear whether these enzymes are involved in downstream of LRR-fungal interactions or if parallel interactions could be involved in species-specific recognition.

In summary, metatranscriptomic analyses show that *Suillus* and *Pinus* exploit a conserved communication system between symbiotic fungi and their hosts (the "symbiosis tool kit" described by Kohler, et al. [10]). We can envision the following scenario leading to mycorrhiza development between compatible *Suillus-Pinus* species pairs: 1) During early stages of mycorrhizal initiation, *Suillus* spp. interact with *Pinus* roots via small secreted proteins and host-specific G-protein signaling; 2) At the same time, the *Pinus* host expresses unique sets of plant receptors (e.g. LRR-proteins) that allow the plant to recognize and interact with its EMF; 3) During compatible interactions, plant-fungal recognition is followed by nutrient exchange between the plant and its EMF. Continued adaptation and coevolution between plant/fungal unique genes is predicted to result in different host-specificity outcomes. Altered recognition by the same core system may also result in incompatibility between symbionts.

#### *Suillus* genes associated with incompatibility

In addition to common functions of *Suillus* that regulate the host-specific recognition across the pairs, comparative transcriptomic analysis also identified genes that displayed a distinctive transcriptional response to incompatible hosts. Those not only include transcripts that are absent in compatible pairings (e.g. twin-arginine translocation (tat) pathway, GPI anchored protein, fungal LRR-domain proteins, phosphatase, alpha/beta hydrolase, and pectin lyase) but also genes with three to over 6,000x significantly higher gene count in response to incompatibility (e.g. small-secreted proteins and membrane protein/receptor) (S1 Dataset). Protein domain analysis provides additional evidence for the role of these genes in plant recognition (S9 Fig), indicating active communication by *Suillus* with both compatible and incompatible hosts. Though mycorrhizae are not established during incompatible interactions (Fig 1A and S1 Fig), gene products associated with incompatible response suggest that *Suillus* communicates with its pine host. Further analyses of the initial stages of symbiosis are needed to identify more “unique genes” involved in host-recognition, including transiently expressed genes during early stages of ectomycorrhiza formation.

#### *Suillus* host specificity and compatibility with white pines

Our results confirm and extend earlier reports of host-range and specificity for *Suillus* spp. that fruit under white pine forests [24,25,47]. Two EMF species we tested (*S*. *decipiens* and *S*. *hirtellus*) showed broad host compatibility and were able to form ectomycorrhizae with all 10 different *Pinus* spp. (Fig 1B). *S*. *decipiens* and *S*. *hirtellus* are reported to fruit under loblolly pine (*P*. *taeda*) and other 2-needle pines [24]. An ecological association between *S*. *hirtellus* with *P*. *taeda* is also supported by environmental metagenomic sequences from other pine forest soils which were able to detect *S*. *hirtellus* within the soil metagenomic community [14,15]. In contrast, *S*. *granulatus*, *S*. *spraguei* and *S*. *americanus* formed abundant ectomycorrhizae only with white pine hosts (*P*. *strobus* and *P*. *monticola*) and were less compatible or else failed to form ectomycorrhizae with hard pines (Fig 1B). All three specialist *Suillus* species were also detected under white pine sites using next generation amplicon sequencing [15], though at least one species (*S*. *spraguei*) was also detected under jack pine forests (*P*. *banksiana*) (S2 Fig).

EMF host compatibility deduced from laboratory experiments may not always reflect compatibility under field conditions [23,48]. Many *Suillus* species that fruit under a single pine host in nature may still form mycorrhizae with unrelated hosts. For example, Palm and Stewart [48] were able to experimentally synthesize ectomycorrhizae between several *Suillus* spp. grown with two different pine hosts (*P*. *banksiana* and *P*. *strobus*), though not every combination was successful. Both of these pines belong to different sections of the genus Pinus (Trifoliae and Qunquefoliae) that diverged 85 MYA ([49], suggesting that other environmental and biological factors also contribute to 'ecological specificity' between *Suillus* spp. and their hosts [23]. Our findings suggest that specialization of *Suillus* spp. on white pines at least is under genetic control, and involves mutual signaling between EMF and plant host.

Detection of fungal transcripts during compatible and incompatible *Suillus-Pinus* pairings suggests that mycorrhizal compatibility is not solely due to failure of basidiospores to germinate, but is the product of bidirectional communication between mycorrhizal fungi and their plant host. Our results also shed some light on the process of host-switching and adaptation by *Suillus* with different Pinaceae hosts. Phylogenetic studies of Suillus reveal that host specificity with white pines has arisen independently several times [27]. For example, *S*. *decipiens* and *S*. *spraguei* are each sister clades within the *Suillus* genus, yet they exhibit different levels of host specificity with different sections of the genus *Pinus*. Similar patterns of white-pine specialization among unrelated EMF suggest that host specificity is likely the result of very recent genome evolution targeting similar genes for mycorrhizal compatibility. Although our study did not examine intraspecific variation that is known to occur in many *Suillus* species [50, 51], our use of genetically diverse basidiospores from separate fruit-bodies as inoculum (instead of a pure-culture of a single fungal strain) reflects the way most pines are colonized in nature, and suggests that host-specificity in this instance is largely due to species-level characteristics (instead of within-population variation).

#### Host specificity of pathogenic fungi vs. EMF

The ability of individual fungal strains to recognize specific plant hosts depends upon specific genes that distinguish compatible fungi from closely related fungal taxa. Results from previous studies on host-specificity in plant-pathogens [52,53] and mutualists [29] suggest that all fungi utilize common molecular mechanisms to recognize compatible/incompatible host plants [12]. The primary gene group involved in this mechanism are the SSPs, key signaling effectors that target the plant apoplast or cytosol, which are recognized by LRR receptors of host plants (host/non-host determination) [1,7]. Successful activation by fungal SSPs results in modulation of plant defenses (compatibility determination), and reprogramming of the host cell metabolism to provide plant nutrients to the fungal symbiont [54]. Similar to biotrophic and nectrotrophic plant pathogenic fungi, ectomycorrhizal fungi appear to employ SSPs to initiate interactions with their plant hosts. Secretion of SSP by biotrophic pathogens can alter SA-triggered immune system [52]; in contrast to EMF, they may primarily target the JA/ET-mediated immunity of their host plants [2;39, this study]. Moreover, plants can trigger a hypersensitive response (localized plant cell death) in response to the SSPs secreted by the biotrophic fungi. We have also observed partial necrosis of root tissues during incompatible *Suillus*-*Pinus* pairings (Fig 2), which may be similar to the hypersensitive response, though additional functional studies are needed to test this hypothesis.

Unlike interactions between pathogens and plants that ultimately lead to host cell degradation or death, mutualistic fungi such as EMF must interact with and help to maintain health with cells of their host plant. Thus, the “symbiotic tool kit” for fungal mutualisms must be very different from the “pathogenic tool kit”. The common genes identified from these studies suggesting that over 3,500 key genes that are required for symbiosis between *Suillus* with *Pinus* (Fig 5A).

#### Analysis of EMF mycorrhizae using RNA-seq

In this study we demonstrated how comparative metatranscriptomics enables in-depth exploration of key genes involved in ectomycorrhiza establishment between specific plant-fungus species pairs. These strategies employing next generation sequencing of metatranscriptomes and *de novo* gene assembly offers a practical solution for the study of plant-fungal interactions in other plant-fungal systems where reference genomes may be unavailable.

## Materials and Methods

### Field study and *Suillus*-*Pinus* pair bioassay

To study the distribution of *Suillus* in natural Pinaceae forest soils, next generation sequencing was conducted to identify fungal operational taxonomic units (OTUs) of *Suillus* from the soils collected in Pinaceae forests across the North America. Technical details and data source to generate S1 Fig can be found in Talbot et al. [15]. For mycorrhizal plant bioassays, seeds of different *Pinus* species were purchased from Sheffield’s Seed Co., Inc. (Locke, NY) (see S3 Table for detailed description). The seeds were surface sterilized in 10% bleach for 10 min, suspended in sterilized water overnight and stratified at 4°C for different time periods prior to germination. Germinated seedlings were planted in sterilized sand and watered using sterile water. Basidiospores of different *Suillus* spp. were collected as spore deposits from field-collected fruit bodies by placing pilei overnight on wax paper or aluminum foil. Fruit body collection data are given in SI text A6.

A *Suillus-Pinus* pairwise bioassay was conducted using basidiospore inoculations with six-week old pine seedlings. Ten *Pinus* species were crossed with five *Suillus* species for a total of 50 pairwise combinations (replicated three times). Basidiospores (10^6^ spores) were suspended in sterile water with 0.1% Tween-20, and added to sterilized 400 g of autoclaved sand to fill a four inch pot. Seedlings growing in sterile sand (without inoculum) were used as controls for all experiments (and also to check for airborne growth chamber contamination). Seedlings were grown in a growth chamber at 25°C, 80% humidity and fluorescent light at 200 μmol for 12 hours per day. At 180-d post-inoculation, EMF root tips were visualized under a dissection microscope, and percentage of EMF root tips were counted in comparison with bare (uninoculated) root tips.

### Sampling

Root tips were harvested from the bioassay pots at 90-d post-inoculation. From each plant, 10 root tips were collected using forceps, frozen in liquid N_2_ and stored in -80°C for RNA extraction. Four species of *Suillus* (*S*. *americanus*; *S*. *granulatus*; *S*. *spraguei (= S*. *pictus)*; *S*. *decipiens*) and three species of *Pinus* (*P*. *monticola*; *P*. *strobus*; *P*. *taeda*) were grown in all 12 pairwise combinations (each replicated three times). Root tips collected from uninoculated *Pinus* species were also included as controls. The controls included six samples for a total of three species of *Pinus* that were replications for two different seedlings for each species (Table 1).

### RNA preparation, cDNA construction and Illumina sequencing

Total RNA was extracted using CTAB/chloroform extraction and LiCl precipitation method as described [32]. The mRNA samples for RNA-seq analysis were performed using a TruSeq RNA sample preparation kit (Illumina, San Diego, CA). The cDNA libraries were sequenced on the Illumina HiSeq 2000 (Illumina, San Diego, CA) instruments in Duke Center for Genomic and Computational Biology (GCB). Thirteen samples were sequenced using a single lane of Illumina run and generated 38Gb of data. The data generated from four lanes were applied for this study. The raw reads were deposited in the NCBI Short Read Archive (accession no. SRP057033).

### Sequence assembly and annotation

We employed a genome-free assembly method to sort reads representing genes for different rRNA, *Suillus*, *Pinus*, and other genes (S3 and S4 Figs and SI Text A1). The computational workflow for sequence assembly (S3 Fig) was modified after Liao et al. [32]. First, *Suillus* sequence references were generated using the sequencing reads generated from *Suillus* fungal cultures, including *S*. *americanus*, *S*. *granulatus*, *S*. *spraguei* and *S*. *decipiens*. Next, *de novo* assembly was applied using Trinity [34]. The quality of the assembled contigs/unigenes for the four *Suillus* species are listed in S1 Table. The filtered reads (~28 million) were mapped onto four sets of reference sequences using bowtie with default settings (http://bowtie-bio.sourceforge.net/index.shtml), including references of fungal rRNA, 16S rRNA, contigs generated from *Suillus* cultures, and EST database of *P*. *taeda*. Remaining unmapped reads (approximately 3-million) were assembled *de novo* into contigs using Trinity followed by sorting into fungal and plant reads BlastX. Detailed descriptions of bioinformatics and databases used for three steps are included in SI text A1. The numbers of reads belonging to *Suillus*, *Pinus*, rRNA (and others) is shown in S1 Database. Comparative analysis of gene expression was used to evaluate their biological functions. The t-test (P<0.01) was used to identify the genes of *Suillus* in response to their compatible vs. in compatible hosts (Fig 3). A false discovery rate (FDR) of 5% was used to identify highly expressed transcripts with at least 2-fold change for the common and unique genes of *Suillus* and *Pinus* (Figs 4–7).

Transcriptome (EST) databases for *S*. *americanus* (19,123 contigs), *S*. *granulatus* (15,724 contigs), *S*. *spraguei* (18,898 contigs) and *S*. *decipiens* (16,871 contigs) were assembled *de novo* from fungal cultures using RNASeq. Besides the transcriptome references generated in our study (S1 Table), the other reference databases used in this study include: Fungal rRNA (NCBI, UNITE); Bacterial 16S (Ribosomal Database Project, http://rdp.cme.msu.edu); *P*. *taeda* EST database (NCBI). The databases were quality filtered using FASTA within the Galaxy web-based package. Detailed protocols for plant and fungal annotation databases are provided in SI text A2-A4.

## Supporting Information

S1 Table Quality assessment for reference transcriptomes of *Suillus* spp. FASTQ Quality Trimmer v1.0.0 was used to trim and quality filter reads (cutoff for quality scores <28). Suillus strain IDs (in parentheses) provided for RNASeq sample ID (e.g. S6_16) and fungal strain ID (e.g. EM31). Additional information for sample IDs is described in S1 Dataset, Table 1 and S3 Table.
(DOCX)

S2 Table *Pinus* seed stocks used in this study. All seeds were purchased from Sheffield's Seed Co., Inc., Locke, New York, with exception of *P*. *muricata*, which was provided by the Bruns lab, UC-Berkeley.
(DOCX)

S3 Table Origins of *Suillus* collections, cultures and spore prints used in this study. Cultures (tissue isolates) were isolated from fresh fruit bodies on MMN media (same media used for maintaining and storing cultures). For each *Suillus-Pinus* species pair examined (Fig 1B), spore prints from three sporocarps (fruit bodies) were pooled and used to inoculate *Pinus* seedlings. Voucher sporocarp collections of each species are deposited with the Duke University fungal herbarium.
(DOCX)

S1 Fig Heat map showing the distribution of *Suillus* species across 18 North American pine forest plots. OTU frequency (based on the ratio of the counts) of internal transcribed spacer (ITS) sequences of *Suillus* versus other fungal taxa amplified from soil samples using 454 sequencing strategies [12]. Frequency of *Suillus* OTUs shown by gray shading (white indicates no *Suillus* taxa detected). Boxes highlight co-occurrence of *Suillus* OTUs with *P*. *taeda* and other white pines, respectively.
(TIF)

S2 Fig Cross-sections of compatible (Com: *S*. *pictus/P*. *strobus*) versus incompatible (Incom: *S*. *pictus/P*. *taeda*) mycorrhiza pairings. S, fungal sheath, In, interfacial apoplast; HN, Hartig-net; Co, cortical cells (cortex); Ep, epidermis; En, endodermis; X, Xylem.
(TIF)

S3 Fig Computational workflow used for sequence assembly. Detailed descriptions is given in SI text A1. DB = database; D2 = Large subunit (28S) rRNA Divergent domain 2.
(TIF)

S4 Fig Percentages of metatranscriptomic reads attributable to *Suillus*, *Pinus* and other organisms (bacteria and other fungi) from *Suillus* mycorrhizal roots detected by Illumina HiSeq. Total number of reads after quality trimming = 28 million.
(TIF)

S5 Fig Computational workflow used for normalization and unique gene annotation for RNA-Seq. The detailed descriptions are indicated in SI text A2, A3 and A4. Sa, *S*. *americanus*; Sg, *S*. *granulatus*; Ss, *S*. *spraguei*; Pm, *P*. *monticola*; Ps, *P*. *strobus*; Pt, *P*. *taeda*
(TIF)

S6 Fig Gene expression of *Suillus* genes in response to compatible and incompatible mycorrhzal pairings. (A) Principal components analysis of loadings for different *Suillus*-*Pinus* species pairings (*Suillus/P*. *monticola* in blue; *Suillus/P*. *strobus* in red; *Suillus*/*P*. *taeda* in green) based on normalized expression (log10) of *Suillus* genes (average 12,000 contigs per sample). (B) Volcano plots showing expression of *Suillus* genes in response to compatible/incompatible *Pinus* hosts (plotted as log2 fold change versus the –log_10_ of the adjusted p-value). The horizontal axis is the log_2_ fold change between of the mean expression value of *Suillus* genes in different pairs. For each *Suillus* species, genes upregulated in response to different pine hosts are shown for white pines (*P*. *monticola* and *P*. *strobus*, red dots) or *hard pine (P*. *taeda*, green dots). Read counts of individual gene contigs are listed in S1 Dataset. Additional details of the analysis workflow are given in S3 Fig
(TIF)

S7 Fig Comparative gene expression of 231 *Suillus* common genes with shared function expressed during compatible mycorrhizal interactions. The common genes of *Suillus* in S1 Dataset were further analyzed for their relative expression rate (SI text A3). A false discovery rate (FDR) of 5% using Benjamini-Hochberg test was used to identify highly expressed transcripts with at least 2-fold change for the genes of *Suillus* in compatible pairs compared to incompatible pairs, un-inoculated control and the free living mycelium (cultures). The color key shows the relative log2 fold changes of the normalized values.
(TIF)

S8 Fig Volcano plots of differential expression patterns of *Suillus* genes with two different Pinus hosts (*Suillus/P*. *monticola* vs. *Suillus/P*. *strobus*). Dots indicate the expression pattern of an individual *Suillus* gene from *Suillus/P*. *monticola* vs. *Suillus/P*. *strobus* pairs. The data (normalized expression rates using DESeq package) for all genes are plotted as log_2_ fold change versus the –log_10_ of the adjusted p-value. Data were generated based upon average 12,000 contigs. Differentially expressed *Suillus* genes (dots and the numbers of the genes) shown in response to *P*. *monticola* (green) and *P*. *strobus* (purple). Black dots represent genes with no significant difference across the comparisons. Cross-comparative expression of deferential expressed genes was analyzed using Wilcox text [13] package to compare *Suillus/P*. *monticola* vs. *Suillus/P*. *strobus* (n = 3; P<0.01; > 2-fold changes). The counts of contigs are listed in S1 Dataset.
(TIF)

S9 Fig The primary (Amino acid sequence), secondary (Topographical model) and tertiary (Ribbon model) structure of *Suillus* host-specific genes. Panels illustrate 20 examples of *Suillus* genes and their responses to different *Pinus* hosts. The topographical models were predicted using Protter v 1.0 (http://wlab.ethz.ch/protter/start/). For the ribbon model, the helix (pink) and sheet structures (yellow) are shown. The protein tertiary structures were predicted using I-TASSER v 3.0 [14;15;16]. C-score is a confidence score for estimating the quality of predicted models by I-TASSER (calculated based on significance of threading template alignments and the convergence parameters of the structure assembly simulations). C-score is in the range from -5 to 2, where a C-score of higher value signifies a model with a higher confidence. (A) Small-secreted protein (SSP); (B) G-protein coupled receptor like (GPCR-like). Gene Ontology = GO0007186, G-protein coupled receptor signaling pathway.
(TIF)

S10 Fig Principal components analysis of normalized expression rates (log2) of *Pinus* genes for *Suillus*-root samples paired with (A) *P*. *monticola*, (B) *P*. *strobus*, and (C) *P*. *taeda*. Within each panel, dots represent the loading of one pine gene from data sources across four root pairs, including *Pinus/S*. *americanus*, *Pinus/S*. *granulatus*, *Pinus/S*. *spraguei*, *Pinus/S*. *decipiens* (n = 3; Wilcox package [12]; P<0.01). Colored dots (red, blue, black) indicate differentially expressed unique pine genes for the samples paired with one *Suillus* species (labeled in the left side of the graphs) compared to other species of *Suillus* (blue = gene overrepresented; red = gene underrepresented). Black dots showed the expression of pine genes with no significant difference across the comparisons. SA, *S*. *americanus*; SG, *S*. *granulatus*; SS, *S*. *spraguei*; SD, *S*. *decipiens*; Control, un-inoculated roots.
(TIF)

S11 Fig Cross-comparative expression of "unique" genes identified from *Pinus taeda*. Control, un-inoculated control (2-fold changes; FDR<0.05). The annotated genes and their normalized values are listed in S5 Dateset. (A) SA, *S*. *americanus*; SG, *S*. *granulatus*; SS, *S*. *spraguei*; SD, *S*. *decipiens*. (B) Relative expression of top 15 gene groups responsible for incompatibility and absence under compatible interactions.
(TIF)

S12 Fig Compatible vs. incompatible gene expression of *Suillus* for one representative *Suillus*-*Pinus* root pair (Sa/Ps1). In this study, under compatible interactions, 17M reads of *Suillus* were recovered from a compatible pair, however, only around 1.7M reads were recovered under incompatible pairs. To test if the normalizations for the *Suillus* reads for compatible and incompatible treatments are compatible, a representative sample of compatible pairs (Sa/Ps1) was used to compare the expression patterns between original reads (All data) and randomly reduced reads (Subsets). Sequence reads of three subsets (1.7M) were randomly resampled from the original reads (17M), followed by normalization using DESeq package. The BiocGenerics package was used to generate the plot showing that expression patterns of most genes were not significant different from original *Suillus* reads versus the three subsets of reduced reads (blue dots). Only 2 to 3 genes showed significant different in their expression patterns (red dots, P<0.01).
(TIF)

S1 Dataset **Section 1.** Numbers of Illumina RNASeq reads of *Suillus* and *Pinus* genes recovered from root tip samples; Section 2. The number of contigs recovered from root samples; Section 3. Lists of interactomes identified by comparative transcriptomics (the number of unique genes showed in Fig 4A); Section 4. Published studies describing function of plant gene groups associated with plant defense response; Section 5. *Pinus* normalized gene expression; Section 6. Sequence counts for 28S rRNA reads (D2 region) recovered from root samples
(XLSX)
