# Correction: Metatranscriptomic Study of Common and Host-Specific Patterns of Gene Expression between Pines and Their Symbiotic Ectomycorrhizal Fungi in the Genus *Suillus*

**DOI:** 10.1371/journal.pgen.1006575

**Published:** 2017-01-26

**Authors:** Hui-Ling Liao, Yuan Chen, Rytas Vilgalys

In the Funding section, one of the grant numbers from the funder National Science Foundation is not listed. The correct funding information is as follows: This research is based upon work supported by National Science Foundation Grants DBI-1046052 and DEB-1554181. The funders had no role in study design, data collection and analysis, decision to publish, or preparation of the manuscript.
