# Correction: Metatranscriptomic Study of Common and Host-Specific Patterns of Gene Expression between Pines and Their Symbiotic Ectomycorrhizal Fungi in the Genus *Suillus*

**DOI:** 10.1371/journal.pgen.1007742

**Published:** 2018-10-19

**Authors:** Hui-Ling Liao, Yuan Chen, Rytas Vilgalys

S1 Text is omitted from the list of Supporting Information. It can be viewed below.

## Supporting information

S1 Text (DOCX)
