# Supplementary material for: Metatranscriptomic Study of Common and Host-Specific Patterns of Gene Expression between Pines and Their Symbiotic Ectomycorrhizal Fungi in the Genus Suillus
Source: PLoS Genet. 2016 Oct 13;12(10):e1006348. doi: 10.1371/journal.pgen.1006348 (PMC5065116; doi:10.1371/journal.pgen.1006348)
Supplement: S1 Table — FASTQ Quality Trimmer v1.0.0 was used to trim and quality filter reads (cutoff for quality scores <28). Suillus strain IDs (in parentheses) provided for RNASeq sample ID (e.g. S6_16) and fungal strain ID (e.g. EM31). Additional information for sample IDs is described in S1 Dataset, Table 1 and S3 Table. (DOCX) [file pgen.1006348.s001.docx]

**S1 Table.** Quality assessment for reference transcriptomes of *Suillus* spp. FASTQ Quality Trimmer v1.0.0 was used to trim and quality filter reads (cutoff for quality scores <28). *Suillus* strain IDs (in parentheses) provided for RNASeq sample ID (e.g. S6_16) and fungal strain ID (e.g. EM31). Additional information for sample IDs is described in Dataset S1, Table 1 and S3 Table.

| Sample ID | *S. americanus* (S6_16; EM31) | *S. granulatus* (S9_3; EM37) | *S. spraguei* (S8_7; EM27) | *S. decipiens* (S11_13; EM49) |
| --- | --- | --- | --- | --- |
| No. total reads | 30,321,196 | 31,841,968 | 24,710,538 | 31,609,240 |
| No. filtered reads^1^ | 28,015,666 | 28,777,616 | 22,453,880 | 28,981,020 |
| No. contigs | 19,123 | 15,724 | 18,898 | 16,871 |
| Mean length of contigs (bp) | 995 | 1,124 | 993 | 1019 |
| Max length of contigs (bp) | 9,840 | 9,840 | 9,840 | 9,840 |
| N50 length of contigs (bp) | 1,693 | 2,094 | 1,752 | 1,727 |
| No. contigs ≥ 500 bp | 10,508 | 8,755 | 9,907 | 9,316 |
