# Supplementary material for: Metatranscriptomic Study of Common and Host-Specific Patterns of Gene Expression between Pines and Their Symbiotic Ectomycorrhizal Fungi in the Genus Suillus
Source: PLoS Genet. 2016 Oct 13;12(10):e1006348. doi: 10.1371/journal.pgen.1006348 (PMC5065116; doi:10.1371/journal.pgen.1006348)
Supplement: S2 Table — All seeds were purchased from Sheffield's Seed Co., Inc., Locke, New York, with exception of P. muricata, which was provided by the Bruns lab, UC-Berkeley. (DOCX) [file pgen.1006348.s002.docx]

**S2 Table.** *Pinus* seed stocks used in this study. All seeds were purchased from Sheffield's Seed Co., Inc., Locke, New York, with exception of *P. muricata*, which was provided by the Bruns lab, UC-Berkeley.

| **Pinus species** | **Common name** | **Code** | **Geographic origin** |
| --- | --- | --- | --- |
| *P. taeda* | loblolly pine | ID 090152 | Georgia |
| *P. ponderosa* | western yellow pine; ponderosa pine | N/A | California |
| *P. elliottii* | slash pine | N/A | Georgia |
| *P. contorta latifolia* | lodgepole pine | ID 000140 | Wyoming |
| *P. banksiana* | jack pine; eastern jack pine; gray pine; black pine; Scrub pine; pin gris | ID 870011 | Minnesota |
| *P. resinosa* | red pine; norway pine | ID 041021 | Minnesota |
| *P. muricata* | bishop pine | N/A | California |
| *P. strobus* | eastern white pine | ID 070219 | Minnesota |
| *P. radiata* | monterey pine; radiata pine | N/A | New Zealand |
| *P. monticola* | western white pine | ID 9001 | Mt. Hood NF, OR |
