# Supplementary material for: Metatranscriptomic Study of Common and Host-Specific Patterns of Gene Expression between Pines and Their Symbiotic Ectomycorrhizal Fungi in the Genus Suillus
Source: PLoS Genet. 2016 Oct 13;12(10):e1006348. doi: 10.1371/journal.pgen.1006348 (PMC5065116; doi:10.1371/journal.pgen.1006348)
Supplement: S3 Table — Cultures (tissue isolates) were isolated from fresh fruit bodies on MMN media (same media used for maintaining and storing cultures). For each Suillus-Pinus species pair examined (Fig 1B), spore prints from three sporocarps (fruit bodies) were pooled and used to inoculate Pinus seedlings. Voucher sporocarp collections of each species are deposited with the Duke University fungal herbarium. (DOCX) [file pgen.1006348.s003.docx]

**S3 Table.** Origins of *Suillus* collections, cultures and spore prints used in this study. Cultures (tissue isolates) were isolated from fresh fruit bodies on MMN media (same media used for maintaining and storing cultures). For each *Suillus-Pinus* species pair examined (Fig. 1B), spore prints from three sporocarps (fruit bodies) were pooled and used to inoculate *Pinus* seedlings. Voucher collections of each species are deposited with the Duke University fungal herbarium (DUKE).

| Fungal species | Sample type | Fungal strain ID | collection date | Host | Location |
| --- | --- | --- | --- | --- | --- |
| *S. americanus* | Culture | EM31 | 10 09 2012 | *P. strobus* | Coatesville, PA |
| *S. americanus* | Culture | EM29 | 10 09 2012 | *P. strobus* | Coatesville, PA |
| *S. americanus* | Culture | EM32 | 10 09 2012 | *P. strobus* | Coatesville, PA |
| *S. granulatus* | Culture | EM37 | 07 19 2013 | *P. strobus* | Coatesville, PA |
| *S. spraguei* | Culture | EM27 | 09 26 2012 | *P. strobus* | Durham, NC |
| *S. americanus* | basidiospore | SA0005 | 09 22 2012 | *P. strobus* | Little Switzerland, NC |
| *S. americanus* | basidiospore | SA0010 | 09 22 2012 | *P. strobus* | Little Switzerland, NC |
| *S. americanus* | basidiospore | SA0011 | 09 22 2012 | *P. strobus* | Little Switzerland, NC |
| *S. granulatus* | basidiospore | SG0004 | 09 22 2012 | *P. strobus* | Little Switzerland, NC |
| *S. granulatus* | basidiospore | SG0009 | 09 22 2012 | *P. strobus* | Little Switzerland, NC |
| *S. granulatus* | basidiospore | SG0014 | 09 22 2012 | *P. strobus* | Little Switzerland, NC |
| *S. spraguei* | basidiospore | SS0006 | 09 22 2012 | *P. strobus* | Little Switzerland, NC |
| *S. spraguei* | basidiospore | SS0012 | 09 22 2012 | *P. strobus* | Little Switzerland, NC |
| *S. spraguei* | basidiospore | SS0013 | 09 22 2012 | *P. strobus* | Little Switzerland, NC |
| *S. decipiens* | basidiospore | SD0002 | 08 24 2012 | *P. taeda* | Hillsborough, NC |
| *S. decipiens* | basidiospore | SD0003 | 08 24 2012 | *P. taeda* | Hillsborough, NC |
| *S. decipiens* | basidiospore | SD0008 | 08 24 2012 | *P. taeda* | Hillsborough, NC |
| *S. hirtellus* | basidiospore | SH0007 | 08 24 2012 | *P. taeda* | Hillsborough, NC |
| *S. hirtellus* | basidiospore | SH0015 | 08 24 2012 | *P. taeda* | Hillsborough, NC |
| *S. hirtellus* | basidiospore | SH0016 | 08 24 2012 | *P. taeda* | Hillsborough, NC |
