# Supplementary figures and images for: Metatranscriptomic Study of Common and Host-Specific Patterns of Gene Expression between Pines and Their Symbiotic Ectomycorrhizal Fungi in the Genus Suillus
Source: PLoS Genet. 2016 Oct 13;12(10):e1006348. doi: 10.1371/journal.pgen.1006348 (PMC5065116; doi:10.1371/journal.pgen.1006348)

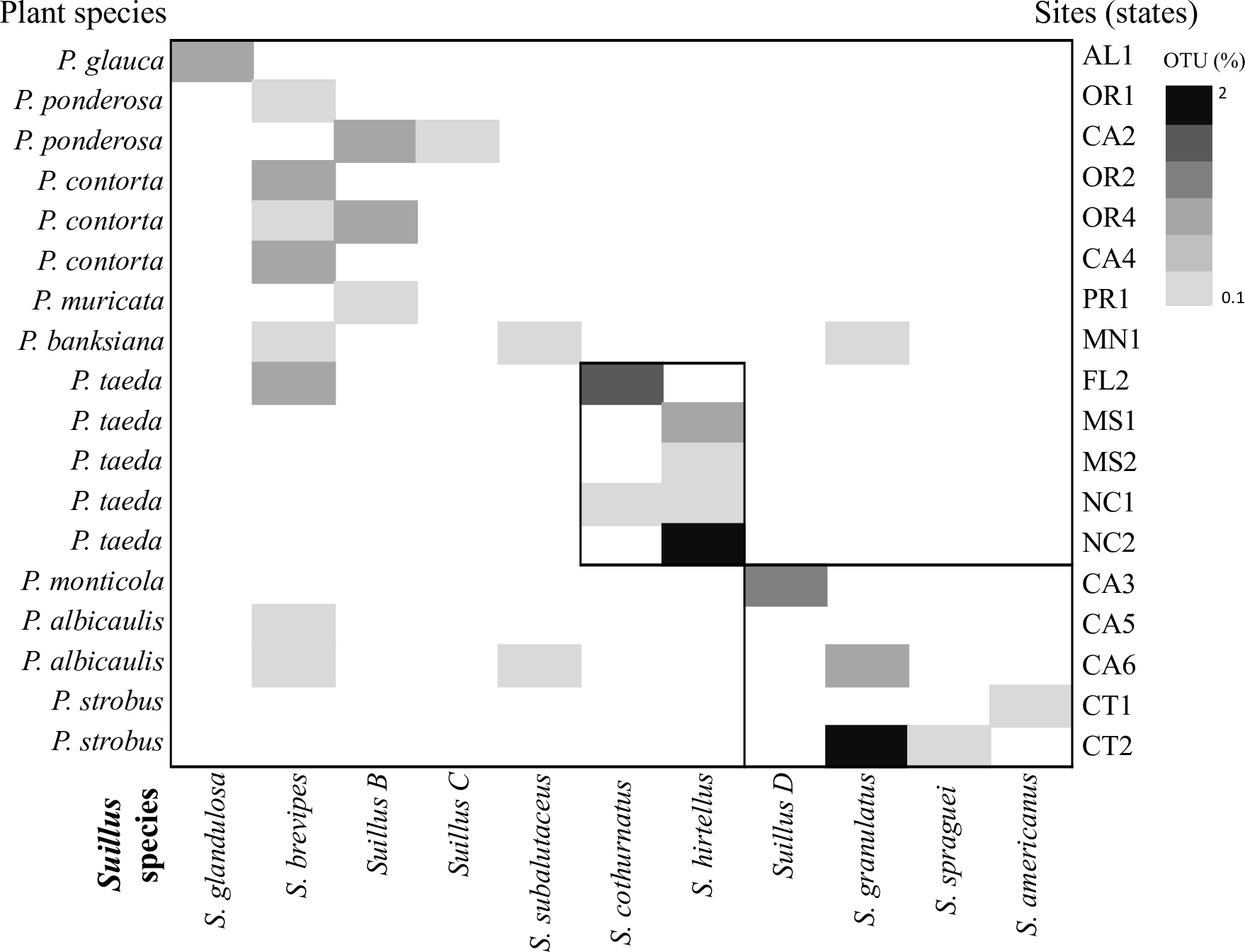

Supplement: S1 Fig — OTU frequency (based on the ratio of the counts) of internal transcribed spacer (ITS) sequences of Suillus versus other fungal taxa amplified from soil samples using 454 sequencing strategies [12]. Frequency of Suillus OTUs shown by gray shading (white indicates no Suillus taxa detected). Boxes highlight co-occurrence of Suillus OTUs with P. taeda and other white pines, respectively. (TIF) [file pgen.1006348.s004.tif]

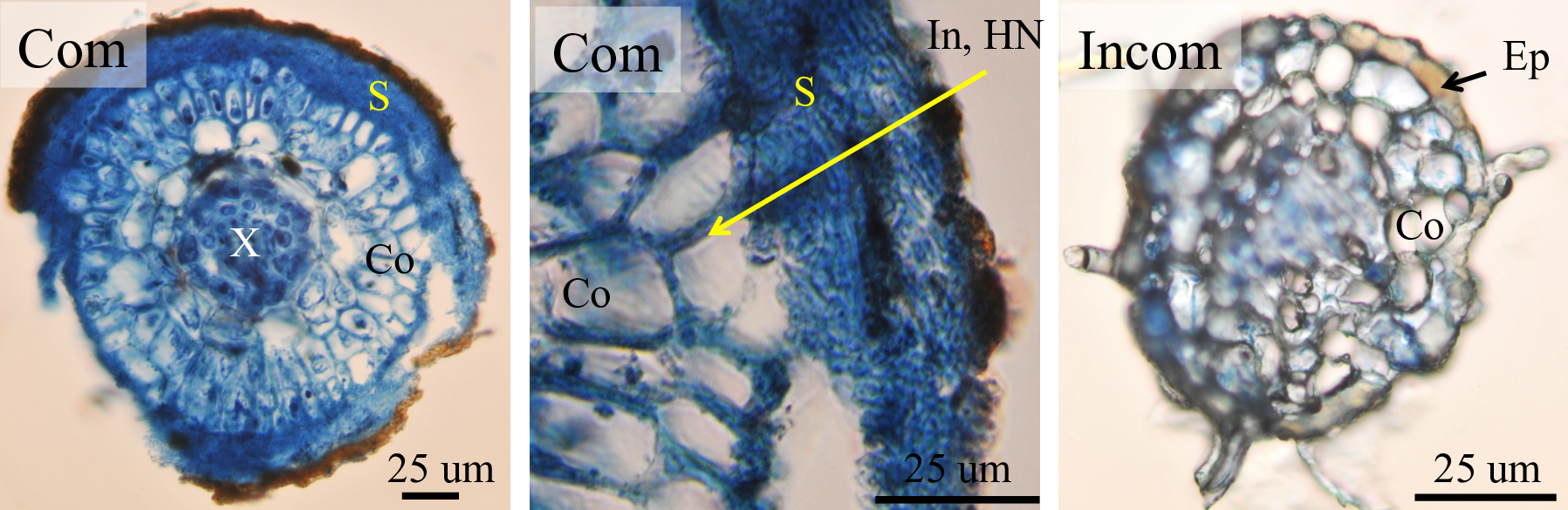

Supplement: S2 Fig — S, fungal sheath, In, interfacial apoplast; HN, Hartig-net; Co, cortical cells (cortex); Ep, epidermis; En, endodermis; X, Xylem. (TIF) [file pgen.1006348.s005.tif]

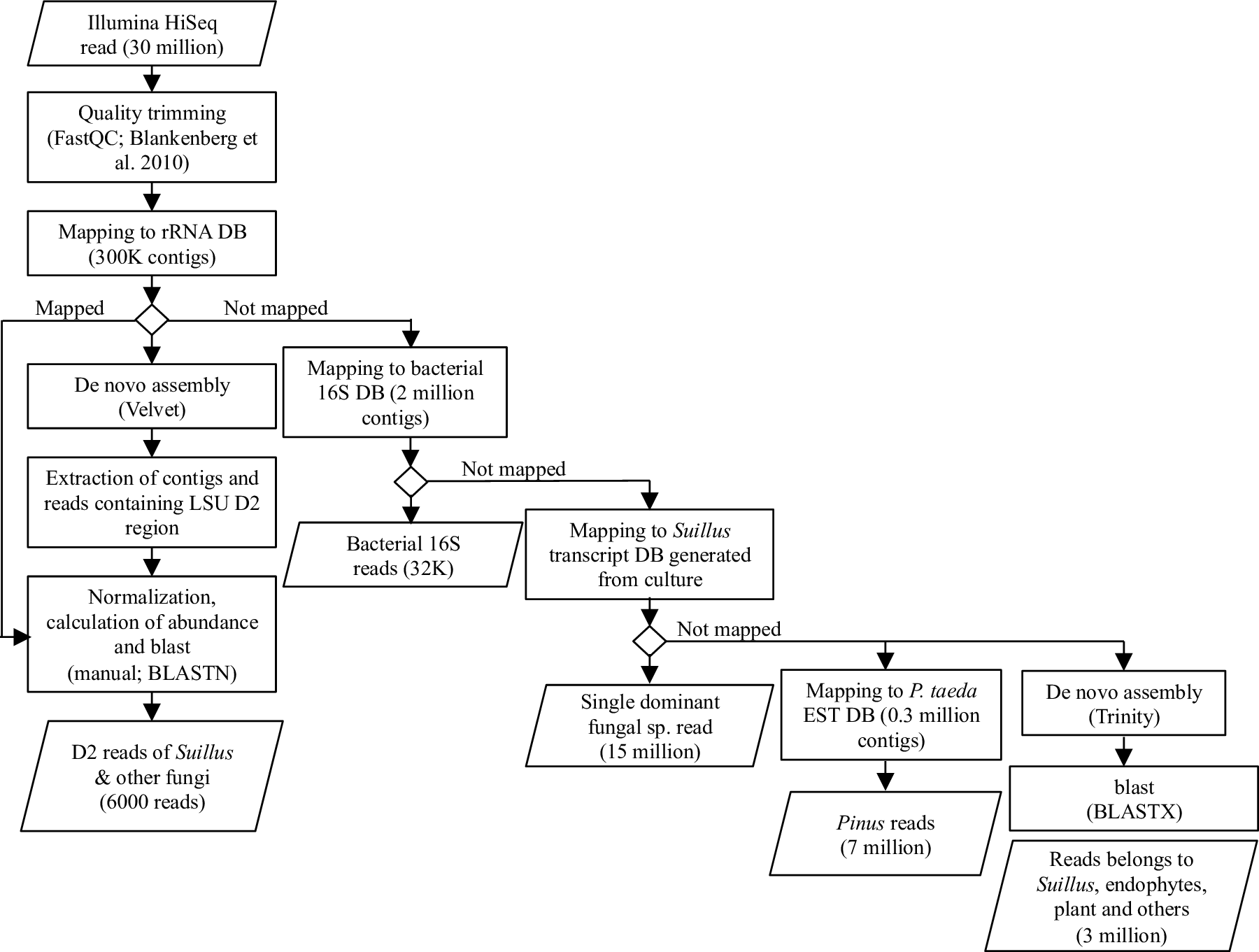

Supplement: S3 Fig — Detailed descriptions is given in SI text A1. DB = database; D2 = Large subunit (28S) rRNA Divergent domain 2. (TIF) [file pgen.1006348.s006.tif]

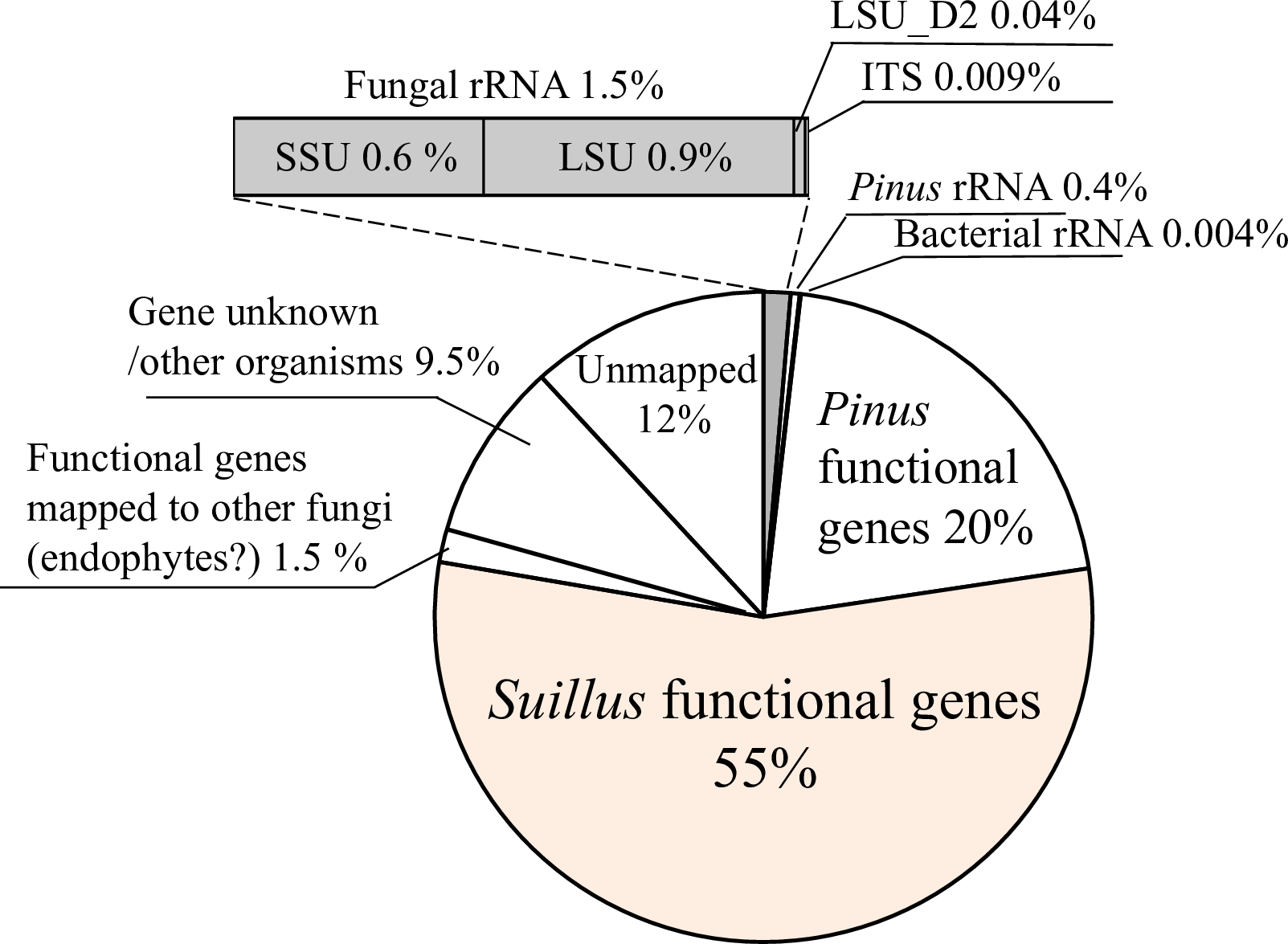

Supplement: S4 Fig — Total number of reads after quality trimming = 28 million. (TIF) [file pgen.1006348.s007.tif]

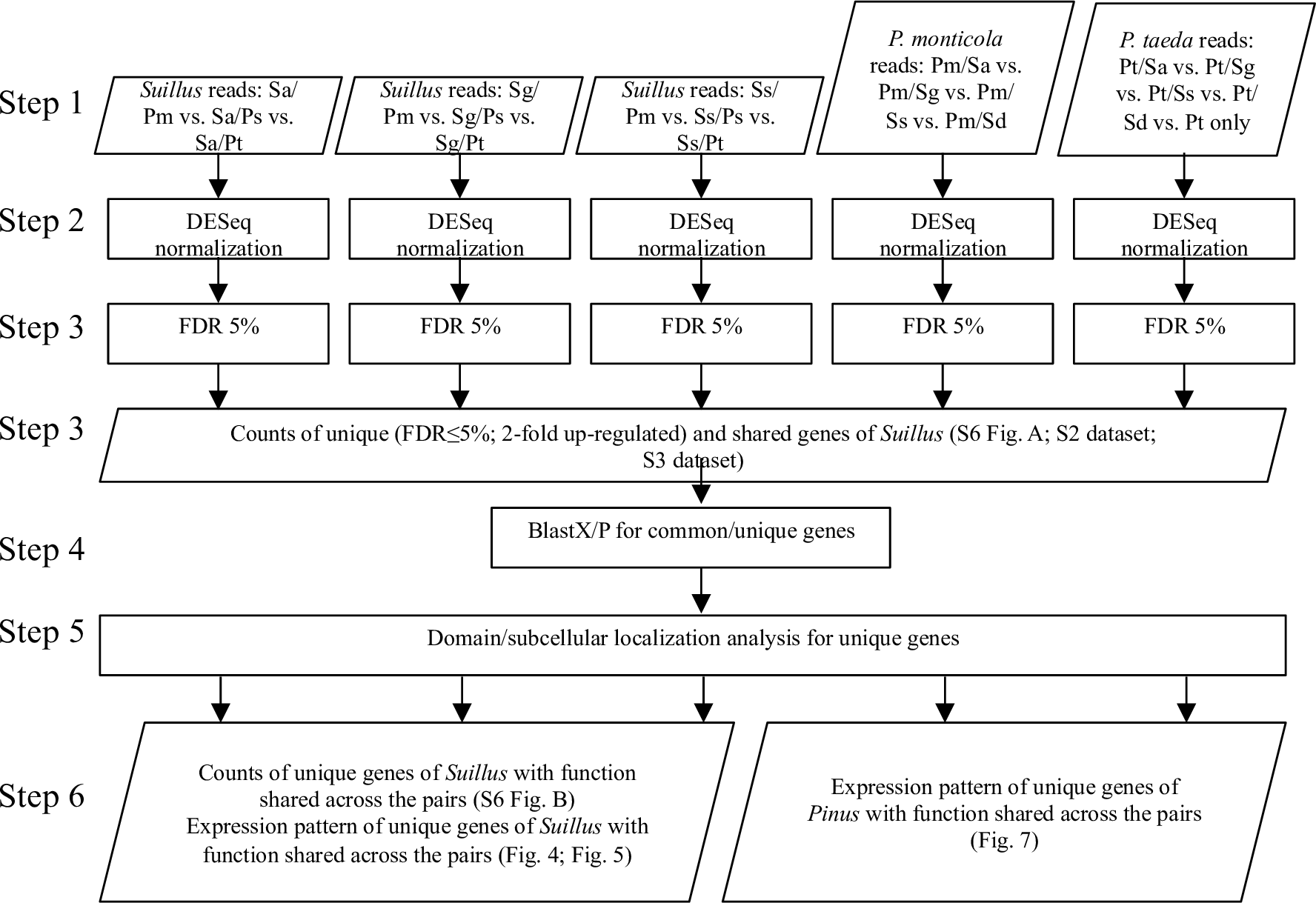

Supplement: S5 Fig — The detailed descriptions are indicated in SI text A2, A3 and A4. Sa, S. americanus; Sg, S. granulatus; Ss, S. spraguei; Pm, P. monticola; Ps, P. strobus; Pt, P. taeda (TIF) [file pgen.1006348.s008.tif]

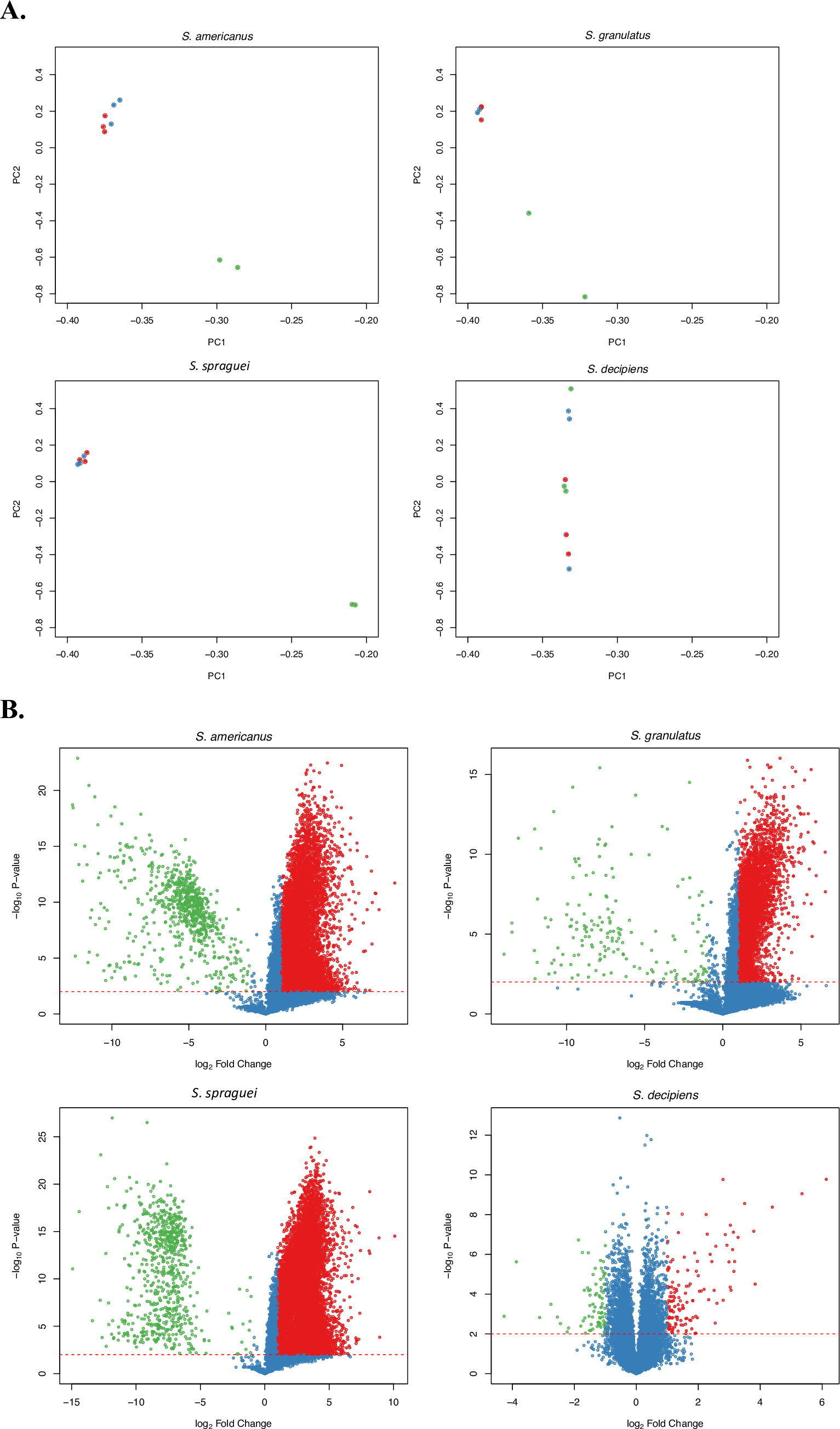

Supplement: S6 Fig — (A) Principal components analysis of loadings for different Suillus-Pinus species pairings (Suillus/P. monticola in blue; Suillus/P. strobus in red; Suillus/P. taeda in green) based on normalized expression (log10) of Suillus genes (average 12,000 contigs per sample). (B) Volcano plots showing expression of Suillus genes in response to compatible/incompatible Pinus hosts (plotted as log2 fold change versus the –log10 of the adjusted p-value). The horizontal axis is the log2 fold change between of the mean expression value of Suillus genes in different pairs. For each Suillus species, genes upregulated in response to different pine hosts are shown for white pines (P. monticola and P. strobus, red dots) or hard pine (P. taeda, green dots). Read counts of individual gene contigs are listed in S1 Dataset. Additional details of the analysis workflow are given in S3 Fig (TIF) [file pgen.1006348.s009.tif]

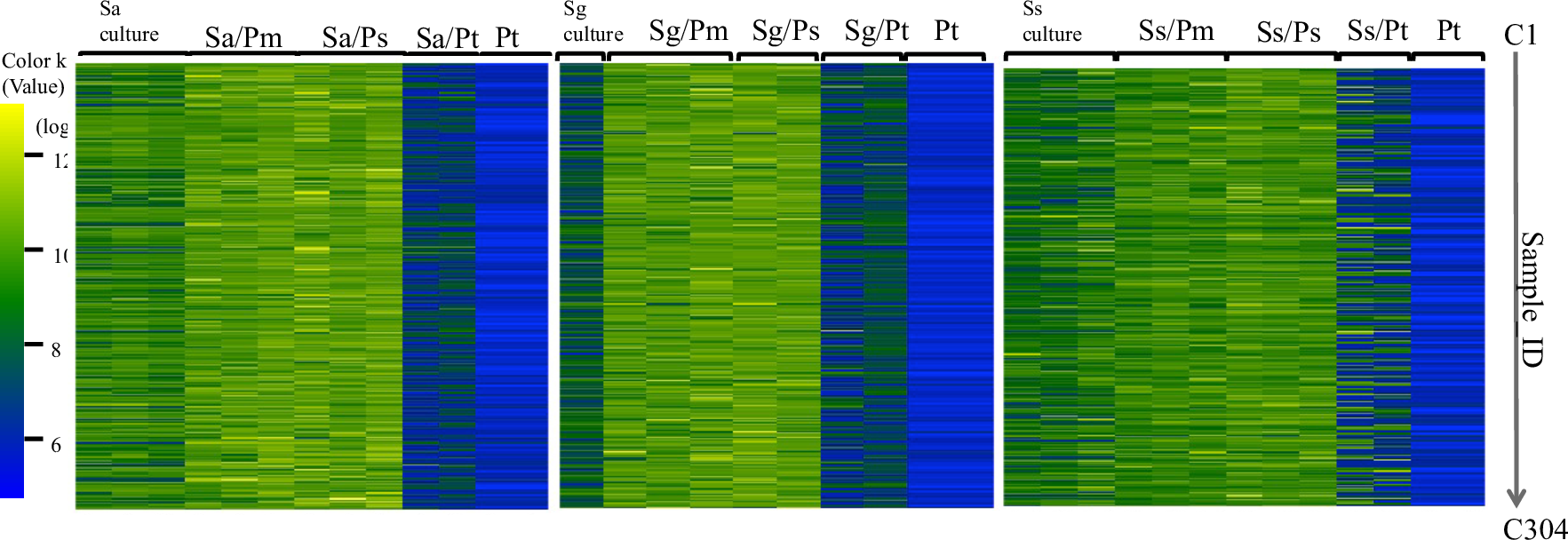

Supplement: S7 Fig — The common genes of Suillus in S1 Dataset were further analyzed for their relative expression rate (SI text A3). A false discovery rate (FDR) of 5% using Benjamini-Hochberg test was used to identify highly expressed transcripts with at least 2-fold change for the genes of Suillus in compatible pairs compared to incompatible pairs, un-inoculated control and the free living mycelium (cultures). The color key shows the relative log2 fold changes of the normalized values. (TIF) [file pgen.1006348.s010.tif]

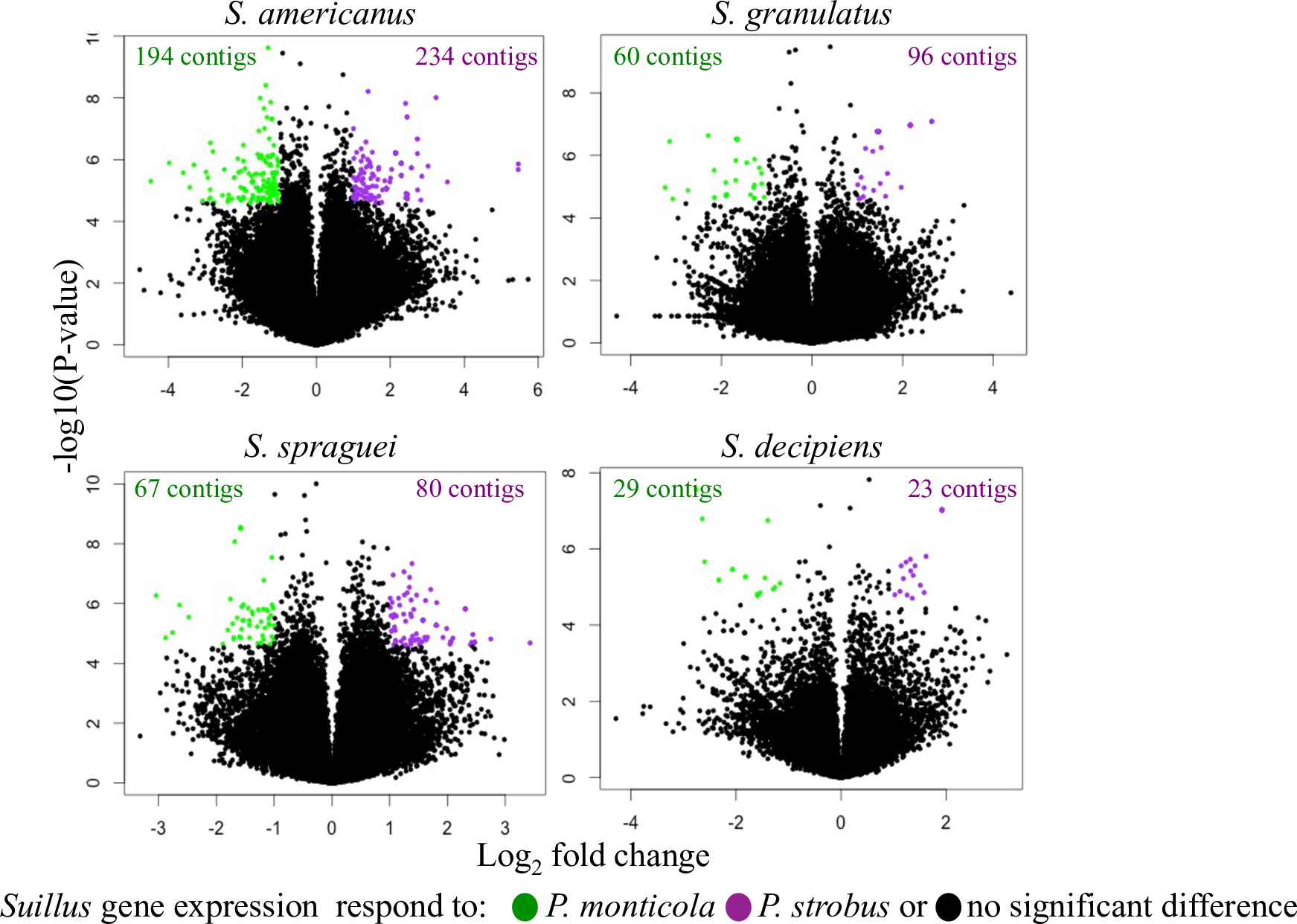

Supplement: S8 Fig — Dots indicate the expression pattern of an individual Suillus gene from Suillus/P. monticola vs. Suillus/P. strobus pairs. The data (normalized expression rates using DESeq package) for all genes are plotted as log2 fold change versus the –log10 of the adjusted p-value. Data were generated based upon average 12,000 contigs. Differentially expressed Suillus genes (dots and the numbers of the genes) shown in response to P. monticola (green) and P. strobus (purple). Black dots represent genes with no significant difference across the comparisons. Cross-comparative expression of deferential expressed genes was analyzed using Wilcox text [13] package to compare Suillus/P. monticola vs. Suillus/P. strobus (n = 3; P<0.01; > 2-fold changes). The counts of contigs are listed in S1 Dataset. (TIF) [file pgen.1006348.s011.tif]

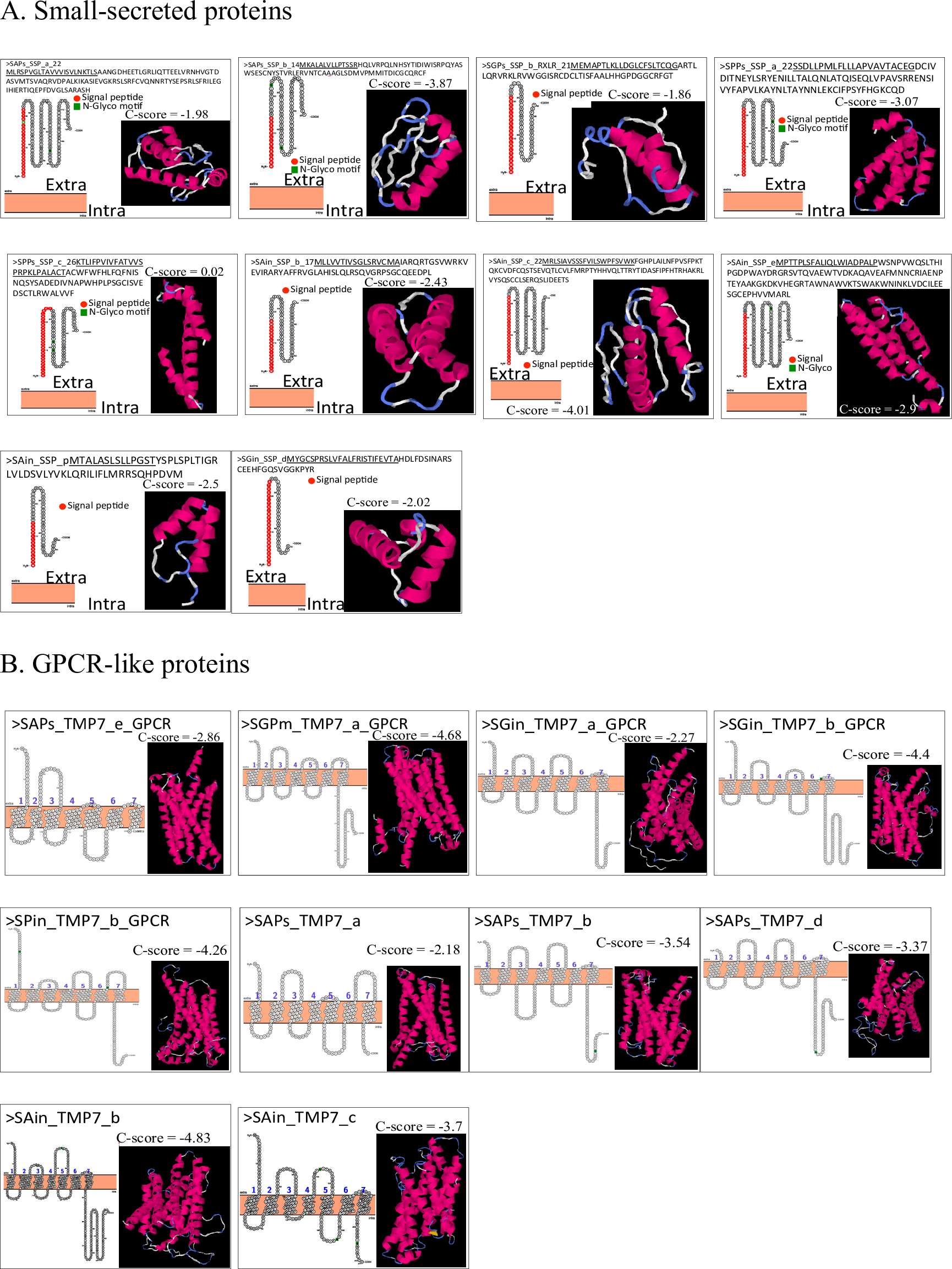

Supplement: S9 Fig — Panels illustrate 20 examples of Suillus genes and their responses to different Pinus hosts. The topographical models were predicted using Protter v 1.0 (http://wlab.ethz.ch/protter/start/). For the ribbon model, the helix (pink) and sheet structures (yellow) are shown. The protein tertiary structures were predicted using I-TASSER v 3.0 [14;15;16]. C-score is a confidence score for estimating the quality of predicted models by I-TASSER (calculated based on significance of threading template alignments and the convergence parameters of the structure assembly simulations). C-score is in the range from -5 to 2, where a C-score of higher value signifies a model with a higher confidence. (A) Small-secreted protein (SSP); (B) G-protein coupled receptor like (GPCR-like). Gene Ontology = GO0007186, G-protein coupled receptor signaling pathway. (TIF) [file pgen.1006348.s012.tif]

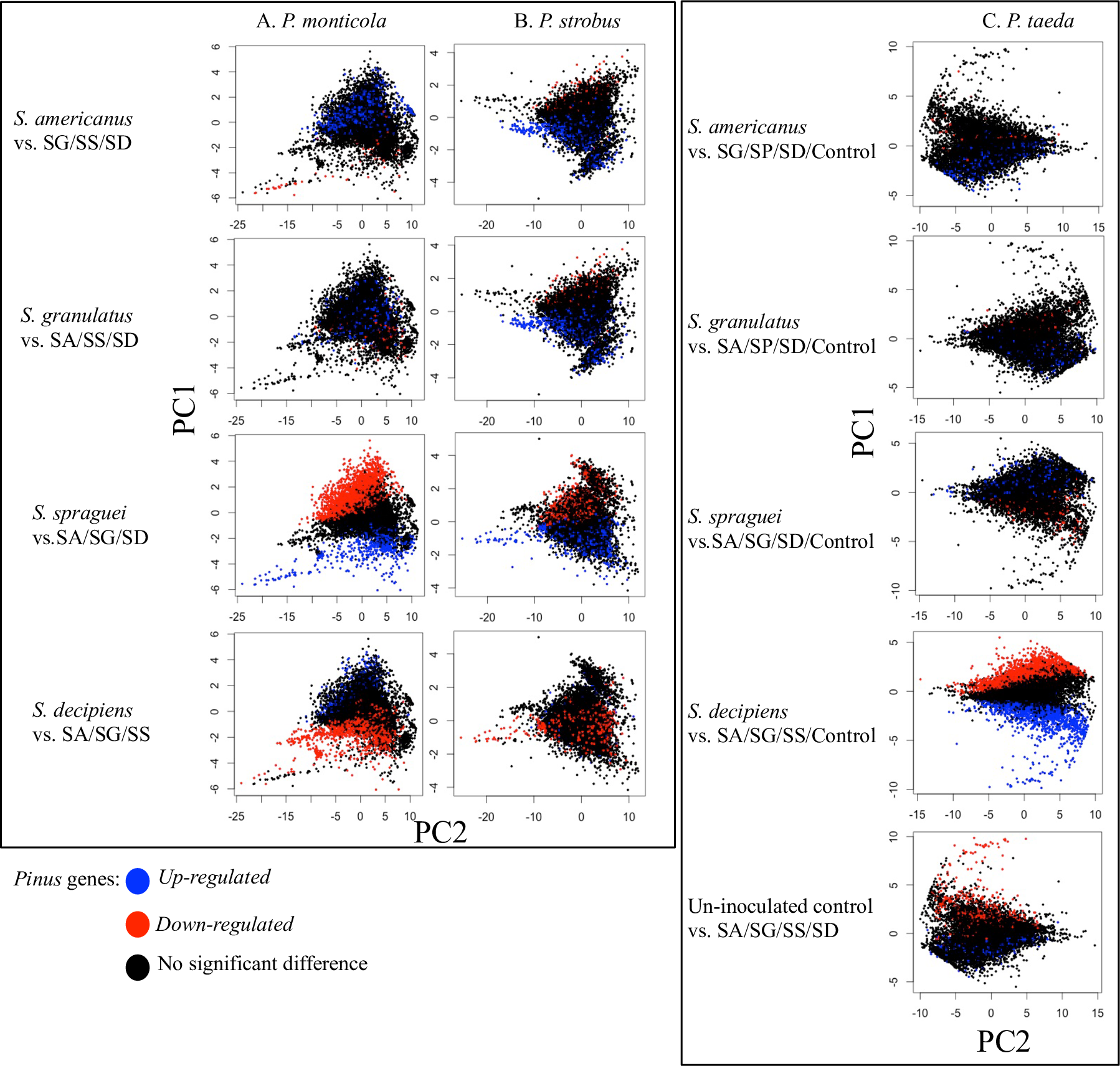

Supplement: S10 Fig — Within each panel, dots represent the loading of one pine gene from data sources across four root pairs, including Pinus/S. americanus, Pinus/S. granulatus, Pinus/S. spraguei, Pinus/S. decipiens (n = 3; Wilcox package [12]; P<0.01). Colored dots (red, blue, black) indicate differentially expressed unique pine genes for the samples paired with one Suillus species (labeled in the left side of the graphs) compared to other species of Suillus (blue = gene overrepresented; red = gene underrepresented). Black dots showed the expression of pine genes with no significant difference across the comparisons. SA, S. americanus; SG, S. granulatus; SS, S. spraguei; SD, S. decipiens; Control, un-inoculated roots. (TIF) [file pgen.1006348.s013.tif]

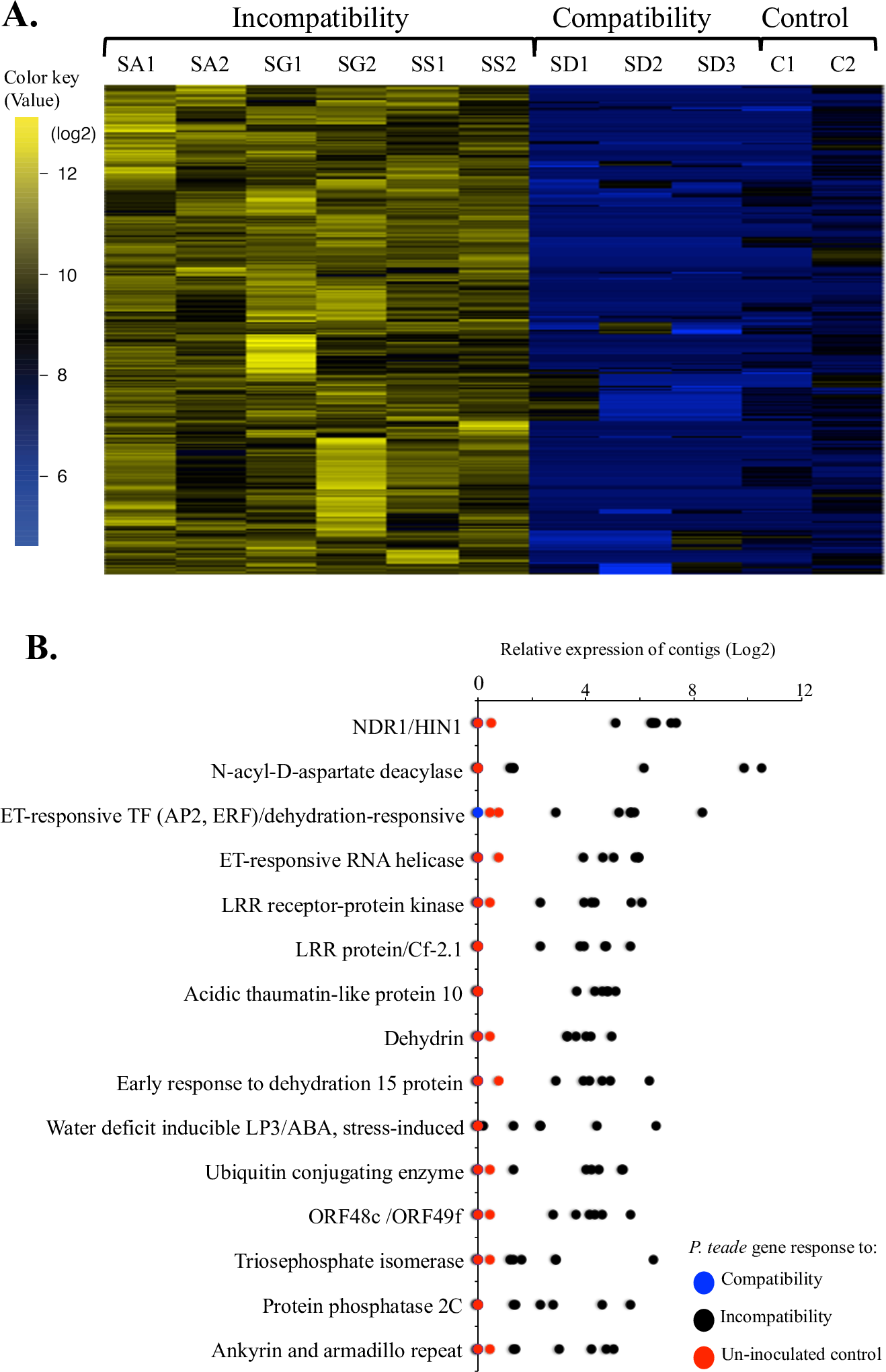

Supplement: S11 Fig — Control, un-inoculated control (2-fold changes; FDR<0.05). The annotated genes and their normalized values are listed in S5 Dateset. (A) SA, S. americanus; SG, S. granulatus; SS, S. spraguei; SD, S. decipiens. (B) Relative expression of top 15 gene groups responsible for incompatibility and absence under compatible interactions. (TIF) [file pgen.1006348.s014.tif]

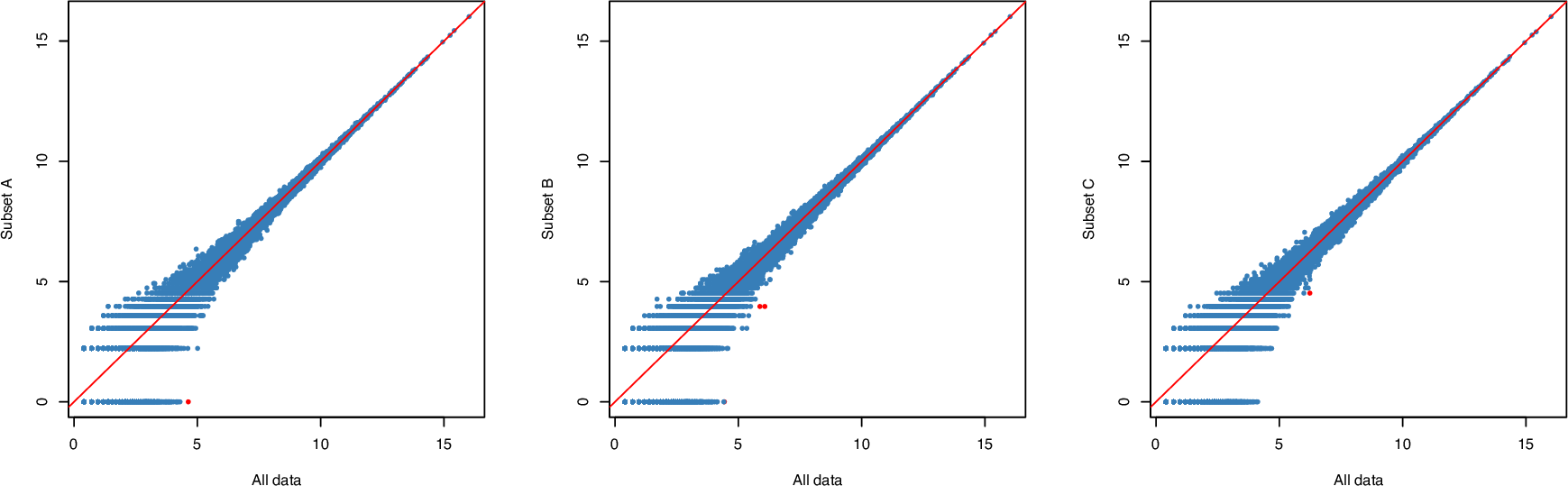

Supplement: S12 Fig — In this study, under compatible interactions, 17M reads of Suillus were recovered from a compatible pair, however, only around 1.7M reads were recovered under incompatible pairs. To test if the normalizations for the Suillus reads for compatible and incompatible treatments are compatible, a representative sample of compatible pairs (Sa/Ps1) was used to compare the expression patterns between original reads (All data) and randomly reduced reads (Subsets). Sequence reads of three subsets (1.7M) were randomly resampled from the original reads (17M), followed by normalization using DESeq package. The BiocGenerics package was used to generate the plot showing that expression patterns of most genes were not significant different from original Suillus reads versus the three subsets of reduced reads (blue dots). Only 2 to 3 genes showed significant different in their expression patterns (red dots, P<0.01). (TIF) [file pgen.1006348.s015.tif]
