# Supplementary material for: Correction: Metatranscriptomic Study of Common and Host-Specific Patterns of Gene Expression between Pines and Their Symbiotic Ectomycorrhizal Fungi in the Genus Suillus
Source: PLoS Genet. 2018 Oct 19;14(10):e1007742. doi: 10.1371/journal.pgen.1007742 (PMC6195257; doi:10.1371/journal.pgen.1007742)
Supplement: S1 Text — (DOCX) [file pgen.1007742.s001.docx]

Table of contents

1. SI Text
   A1. Sequence assembly and RNAseq
   A2. Read normalization and comparisons for root RNAseq
   A3. Identification of common genes identified across *Suillus/Pinus* compatible pairs
   A4. Annotation for the unique genes identified from individual *Suillus/Pinus* pairs
   A5. Transmembrane protein & WD-40 repeat (beta-transducin repeat)
   A6. Biomaterial collection for fungal inoculum and culture
   A7. RNA extraction & sequencing from *Suillus* culture
2. References

**A1. Sequence assembly and RNAseq**

To assess the *Suillus* transcriptome in EMF-root tips, a combination of reference-based assembly using the *Suillus* transcriptome datasets and *de-novo* assembly was performed as described by Liao et al. (2014). Cultures of known *Suillus* spp. were used to generate references for assembly and annotation, including *S. americanus*, *S. granulatus*, *S. spraguei* and *S. decipiens*. Reads that passed quality control were used for *de novo* assembly using Trinity [1]. The quality of the assembled contigs/unigenes for the four *Suillus* species is shown in Table S1. The assembled reference contigs were used to map Illumina HiSeq RNA reads generated from EMF root tips using Bowtie (S3 Fig). (Complete annotations of the contig sets generated from *Suillus* cultures will be presented elsewhere together with genome sequences). Hybrid methods combining reference-based and *de novo* assemblies were performed to sort reads representing fungal and plant genes (Fig 2; S3 & S4 Fig; S6 dataset). The filtered reads (~28 million reads) were mapped onto reference sequences using Bowtie using default settings (http://bowtie-bio.sourceforge.net/index.shtml). Remaining unmapped reads (approximately 3-million) were assembled into contigs using Trinity [1], and sorted into fungal and plant contigs using BlastX. For identification of *Pinus* genes, annotated sequences of coding genes from 14 woody and herbaceous plant species (*Pinus taeda, Picea sitchensis, Populus trichocarpa, Populus euphratica, Amborella trichopoda, Jatropha curcas, Glycine max, Vitis vinifera, Zea mays, Ricinus communis, Arabidopsis thaliana, Beta vulgaris, Gossypium raimondii, and Sorghum bicolor* were downloaded from NCBI to use used as references for annotation.

**A2. Read normalization and comparisons for root RNAseq.** Up to 10 times as many *Suillus* reads were recovered from compatible versus incompatible mycorrhizal roots (17M vs. 1.7M reads on average). To correct for the impact of different size data sets on inferred gene expression, the DESeq 1.14.0 package (using R program with default settings) was used to normalize the read counts mapped to *Suillus* or *Pinus* transcripts [2]. For comparative transcriptomics, read counts were adjusted to 1.2M reads/replicate sample. S1 Dataset lists replicates used for comparative transcriptomes. A representative sample of compatible pairs (Sa/Ps1) was used to compare the expression patterns between original reads (17M) and randomly reduced read counts (1.7M). Similar gene expression patterns were observed between original *Suillus* reads versus three subsets with reduced numbers of reads (S12 Fig). FDR tool 1.2.15 package [3] or Benjamini-Hochberg test were used to identify contigs with significantly different expression (S12 Fig).

We developed a pipeline (S5 Fig) to identify the sets of genes that were differentially expressed in response to compatible and incompatible *Suillus*/*Pinus* pairings. Sequence reads for *Suillus* and *Pinus* were recovered as described in S3 Fig, and mapped to the databases of *Suillus* or *Pinus* respectively. Read counts aligned to individual contigs were used to estimate abundances, followed by normalization of read counts for each Suilus-Pinus paired data sets using the DESeq package. For example, the data sets of *Suillus* collected from Sa/Pm, Sa/Ps and Sa/Pt were normalized and compared (FDR, 5%, 2-fold up-regulated) to identify the *Suillus* genes that were up-regulated only in a pair combination (unique genes) or commonly shared across the compatible pairs of four white-pine associated *Suillus* species (common genes); additional details are described in SI text A3). *Suillus* reads were compared between compatible versus incompatible *Suillus/Pinus* roots; additional transcriptome reads from cultures were used for our comparison to confirm identify of common/unique genes of *Suillus* that were up regulated in associated with fungal-root interaction (Examples shown in S7 Fig.).

T-test (P<0.01) was used to identify differences in gene expression in response to their compatible vs. incompatible hosts (S5-S8 Fig). A false discovery rate (FDR) of 5% was used to identify highly expressed transcripts with at least 2-fold change for the common and unique genes of *Suillus* and *Pinus* (Fig 4-Fig 7).

**A3. Identification of common genes across *Suillus/Pinus* compatible pairs.**

BlastP (>50% nucleotide identity with >50% query coverage) was applied to identify 231 shared (common) *Suillus* genes expressed by all four species of *Suillus* (Sa/Sg/Ss/Sd) when paired with two compatible hosts (Pm/Ps) (S3 Dataset_A).

Homology of genes assembled by de-novo approaches was further confirmed by phylogenetic analysis against related genes using BLASTp searches against JGI Mycocosm web portal using published genomes for *Suillus brevipes* and *S. luteus*, as well as through BLASTp searches against the NCBI protein database (for other mycorrhizal fungi).

**A4. Annotation of unique genes identified from individual *Suillus/Pinus* pairs.** 74 to 571 “unique genes” of *Suillus* and 30-60 “unique genes” of *Pinus* were identified from nine individual *Suillus/Pinus* root pairs respectively (Fig. 4; S3 dataset). For these genes, amino acid prediction from nucleotide sequences was performed using ExPASy (<http://web.expasy.org/translate/>). Over 60% of expressed genes from *Suillus* perspectives were assembled to full-length (S3 Dataset). Transcripts with relatively lower expression levels were only partially assembled (due to the unevenness of sequencing coverage).

A combination of BlastX/BlastP (>50% nucleotide identity with >70% query coverage) [4], GO [5], KEGG [6] and KOG [7] packages was used for gene annotation. NCBI [8] and Myhits were applied for production of conserved domains and motifs (<http://www.ncbi.nlm.nih.gov/Structure/cdd/wrpsb.cgi>). For BlastX/BlastP calls, the annotated sequences of coding genes from 30 species of ectomycorrhizal and wood-rotting fungi were downloaded from NCBI to use as references for fungal gene annotation using BlastX. Similarly, annotated sequences of coding genes from 14 woody plant species used as references for *P. taeda* gene annotation. The same NCBI-databases were used to sort un-mapped fungal and plant genes from root samples (See above). The results of annotation data are shown in S3, S4 & S5 Dataset. Default parameters were used for all bioinformatics analyses.

For every *Suillus/Pinus* species pair that we tested, most of the uniquely expressed contigs (over 60% of “unique genes”) were annotated as having unknown function. Several packages for domain annotation were used to identify the subcellular locations of genes with functional unknown. For domain analysis, EMBL-EBI (<http://www.ebi.ac.uk/Tools/pfa/phobius/>), Signal-3L (<http://www.csbio.sjtu.edu.cn/bioinf/Signal-3L/>), Signal P v4.1[9], and TMHMM v2.0 (<http://www.cbs.dtu.dk/services/TMHMM/>) were used for the prediction of signal-peptides and transmembrane helix domains.;). ER retention signal_ScanPrositeTool (http://prosite.expasy.org/scanprosite/) and Euk-mPLoc 2.0 (http://www.csbio.sjtu.edu.cn/cgi-bin/EukmPLoc2.cgi) was used to identify the subcellular localization of contigs. Small secreted proteins were defined as having (a) size smaller than 300 amino acid (detected manually), (b) signal peptide predicted at the N-end (Signal-P v4.1), (c) extracellular location (Euk-mPLoc 2.0), (d) no transmembrane domains (Euk-mPLoc 2.0, TMHMM v 2.0, EMBL-EBI and Signal-3L), (e) no ER retention motifs (ER retention signal_ScanPrositeTool). Using this approach, we were able to assign most contigs of unknown function as small-secreted proteins or transmembrane proteins.

In addition, we evaluated sub-localization and function of the “unique genes” from their primary, secondary and tertiary characteristics. The predicted amino acid sequences of interactomes that were described below are listed in S3 Dataset. Examples of the images for some of the predicted proteins are shown in S9 Fig.

**A5. Transmembrane protein & WD-40 repeat (beta-transducin repeat).**

The transmembrane proteins that mediate signal perception at the interface between plant and the fungus are of key interest. Around 30 contigs (>15% of *Suillus* interactome) that code for transmembrane domains were identified. In addition to G-protein coupled receptors (GPCRs), most of the predicted proteins are of unknown function (BLASTp, e-value <10^-3^). For the secondary characteristics, the diverse predicted proteins contain a range of 1-14 transmembrane domains (S3 Dataset). Even proteins with same number of transmembrane domains were found to differ in length and the structure of their predicted extracellular regions (S9B Fig).

WD-40 repeat (beta-transducin repeat) proteins usually contain short (~40) amino acid motifs and a Trp-Asp (W-D) near their C-terminal. The *Suillus* WD40 repeats code for a 4-16 bladed beta-propeller (data not shown). Some of the sequences form a circularized beta-propeller structure (G-beta like). Some of the sequences also form the beta-propellers and alpha-helix domains (G-alpha or G-binding kinase like). The repeated WD40 motifs may act as a site for protein-protein interaction to form transient complexes. The predicted functions of *Suillus* WD40 proteins include G-beta, GTPase, serine/threonine protein kinase, GTP-bing HET protein, and functional unknown (S3 Dataset), indicating their function associated with G-protein signaling.

**A6. Biomaterial collection for fungal inoculum and culture** *Suillus basidiocarp collection*: Fresh *Suillus* basidiocarps (fruit bodies) were collected from *Pinus* forests. Spore prints were obtained from pilei placed overnight onto wax paper or aluminum foil. Details of collections and cultures used for this study are given in Table S3. Spore prints were stored at -20 °C until use. *Suillus* cultures were isolated as tissue isolates from basidiosporocarp tissues. To culture *Suillus*, approx 0.1-0.5 mg of fungal tissue from the interior of fresh sporocarps was collected using sterile forceps and placed onto MMN medium containing 50 mg/ml streptomycin [10].

**A7. RNA extraction & sequencing from *Suillus* cultures.** RNA extracted from fungal cultures was used to generate a reference for RNAseq. To prepare fungal cultures for RNA extraction, approx 10 mg mycelium from MMN plate cultures was macerated in 1-ml of sterile distilled water and inoculated into an Erlenmeyer flask containing 50 ml liquid MMN liquid medium. After eight days of growth at 25 °C with shaking (100 rpm), fungal mycelium was collected by filtration over Miracloth (Calbiochem, Darmstadt, Germany), washed using sterile distilled water, frozen in liquid nitrogen and ground using a sterilized mortar and pestle. RNA was extracted using RNeasy plant mini kit (Qiagen). The methods for RNA quantification, qualification, cDNA library construction and Illumina RNA sequencing are described in the material and methods in the text. FastQC was used to check the quality for the raw reads generated on the Illumina HiSeq 2000 (Illumina, San Diego, CA). FASTQ Quality Trimmer v1.0.0 was used to trim and quality filter the raw reads generated with cutoff with quality scores<28) [11].

**B. References**

1. Anders S, Huber W. Differential expression analysis for sequence count data. Genome Biol. 2010;11:R106.
2. Methodological description: Strimmer, K. 2008. A unified approach to false discovery rate estimation. [BMC Bioinformatics 9: 303](http://dx.doi.org/10.1186/1471-2105-9-303).
3. Tao T**.** Standalone BLAST Setup for Unix. In: BLAST Help [Internet]. Bethesda (MD): National Center for Biotechnology Information (US); 2008-. Available from: <http://www.ncbi.nlm.nih.gov/books/NBK52640/>, 2010.
4. Ashburner M, et al. Gene Ontology: tool for the unification of biology. The Gene Ontology Consortium. Nature Genet*.* 2000;25:25-29.
5. Kanehisa M, Goto S, Sato Y, Furumichi M, Tanabe M. KEGG for integration and interpretation of large-scale molecular datasets. Nucleic Acids Res. 2012;40:D109-D114.
6. Tatusov RL, et al. The COG database: an updated version includes eukaryotes. BMC Bioinformatics. 2003;4:41:1-14.
7. Marchler-Bauer A, et al. CDD: a Conserved Domain Database for the functional annotation of proteins. Nucleic Acids Res. 2011;39:225-229.
8. Petersen TN, Brunak S, von Heijne G, Nielsen H. SignalP 4.0: discriminating signal peptides from transmembrane regions. Nature Methods. 2011, 8:785-786.
9. Blankenberg D, et al. Manipulation of FASTQ data with Galaxy. Bioinformatics. 2010;26:1783-1785.
10. Bauer DF. Constructing confidence sets using rank statistics. JASA. 1972;67:687-690.
11. Zhang Y. I-TASSER server for protein 3D structure prediction. *BMC* Bioinformatics. 2008;9:40.
12. Roy A, Kucukural A, Zhang Y. I-TASSER: a unified platform for automated protein structure and function prediction. Nature Protocols. 2010;5:725-738.
13. Roy A, Yang J, Zhang Y. COFACTOR: An accurate comparative algorithm for structure-based protein function annotation. Nucleic Acids Res. 2012;40:W471-W477.
